# Insights into Natural Products from Marine-Derived Fungi with Antimycobacterial Properties: Opportunities and Challenges

**DOI:** 10.3390/md23070279

**Published:** 2025-07-03

**Authors:** Muhammad Azhari, Novi Merliani, Marlia Singgih, Masayoshi Arai, Elin Julianti

**Affiliations:** 1 School of Pharmacy, Bandung Institute of Technology, Jl. Ganesha No. 10, Bandung 40132, Indonesia; mazhari@itb.ac.id (M.A.); novimrl@gmail.com (N.M.); marlia@itb.ac.id (M.S.); 2 Laboratory of Natural Products for Drug Discovery, Graduate School of Pharmaceutical Sciences, Osaka University, 1-6 Yamadaoka, Osaka 565-0871, Japan; araim@phs.osaka-u.ac.jp

**Keywords:** tuberculosis, marine-derived fungi, natural products, antimycobacterial, dormant

## Abstract

Tuberculosis (TB) poses a persistent global health threat exacerbated by the emergence of drug-resistant strains; hence, there is a continuous quest for novel antimicrobial agents. Despite efforts to develop effective therapies, existing treatments require a relatively long duration of therapy to eradicate the pathogen due to its virulence factors, pathogenesis patterns, and ability to enter dormant states. This can lead to a higher risk of treatment failure due to poor patient adherence to the complex regimen. As a result, considerable research is necessary to identify alternative antituberculosis agents. The marine environment, particularly marine-derived fungi, has recently gained interest due to its potential as an abundant source of bioactive natural products. This review covers 19 genera of marine-derived fungi and 139 metabolites, 131 of which exhibit antimycobacterial activity. The integrated dataset pinpoints the fungal genera and chemical classes that most frequently yield potent antimycobacterial hits while simultaneously exposing critical gaps, such as the minimal evaluation of compounds against dormant bacilli and the presence of underexplored ecological niches and fungal genera. Several compounds exhibit potent activity through uncommon mechanisms, including the inhibition of mycobacterial protein tyrosine phosphatases (MptpB/MptpA), protein kinase PknG, ATP synthase and the disruption of mycobacterial DNA via G-quadruplex stabilization. Structure–activity relationship (SAR) trends are highlighted for the most potent agents, illuminating how specific functional groups underpin target engagement and potency. This review also briefly proposes a dereplication strategy and approaches for toxicity mitigation in the exploration of marine-derived fungi’s natural products. Through this analysis, we offer insights into the potency and challenges of marine-derived fungi’s natural products as hit compounds or scaffolds for further antimycobacterial research.

## 1. Introduction

*Mycobacterium tuberculosis*, the causative agent of tuberculosis, was first reported on in March 1882 by Robert Koch, though the history of tuberculosis began thousands of years ago [1]. The WHO Global Tuberculosis Report 2022 revealed 10.6 million cases, with Southeast Asia being the highest contributing number, followed by Africa and the Western Pacific, indicating that most patients were found in developing countries [2]. Factors such as poverty, limited access to healthcare, HIV co-infection, malnutrition, and overcrowded living conditions are thought to underlie these high case numbers. Tuberculosis spreads easily via droplets, so such conditions greatly increase the possibility of local epidemics [3]. Additionally, *M. tuberculosis*’s virulence factors, unique pathogenesis, and capacity to enter a non-replicating (dormant) state are major reasons for the disease’s persistence and the complexity of its therapy, despite the availability of current medications and nationwide prevention strategies [4,5,6]. This phenomenon urges the need to find alternatives with a better mode of action to shorten the duration of therapy and simplify the therapy regimen. Also, the emergence of antibiotic-resistant *M. tuberculosis* strains makes the search for alternative antituberculosis agents more imperative than ever.

Marine organism diversity offers rich opportunities to explore novel and medically promising NPs. Although only a tiny fraction of active MF NPs have been identified, they show higher successful application rates than other natural resources, indicating underexplored yet valuable assets [7]. Among various microorganisms that reside independently or in association with other organisms (such as sponges and coral reefs), fungi occupy most of the marine environment. In 2019, marine-derived fungi (MF) NPs accounted for almost 47% of the total marine-derived NPs reported, indicating that MF could be a promising source for NP research, with diverse biological activities encompassing antifungal, antibacterial, cytotoxic, and other biological activities [8]. This suggests that continuing the study of MF is essential to discover new and potentially beneficial bioactive compounds that can help tackle various global challenges, including infectious diseases.

Marine-derived fungi represent a promising source of antimycobacterial activity because they can be cultivated and, in many cases, genetically manipulated to support gram-to-kilogram-scale metabolite production. This review collates and summarizes secondary metabolites with confirmed antimycobacterial activity that have been isolated from marine fungi recovered from diverse habitats, including coastal sediments, mangroves, sponges, and deep-sea environments. Relevant primary papers were retrieved by prioritizing original reports reporting minimum inhibitory (MIC) or half-maximal inhibitory (IC_50_) concentrations against *Mycobacterium* or validated mycobacterial enzyme targets, with the objective of identifying compounds that warrant further evaluation as lead compounds for alternative antituberculosis development. In this article, we present extensive coverage over the period of 2002–2023, with 131 out of 139 MF NPs exhibiting antimycobacterial properties reported from various MF genera. The compounds are presented in groups based on the common chemical structures, namely, polyketides, peptides, alkaloids, terpenoids, and steroids. We also highlight the novelty of the MF NPs at the time of their being reported as having antimycobacterial properties. The mechanisms of action of some compounds are discussed and the structure–activity relationships of the most potent compounds are also briefly outlined.

## 2. Antimycobacterial Compounds from Marine-Derived Fungi

### 2.1. Marine-Derived Fungi and Their Marine Sources

**Figure 1:**
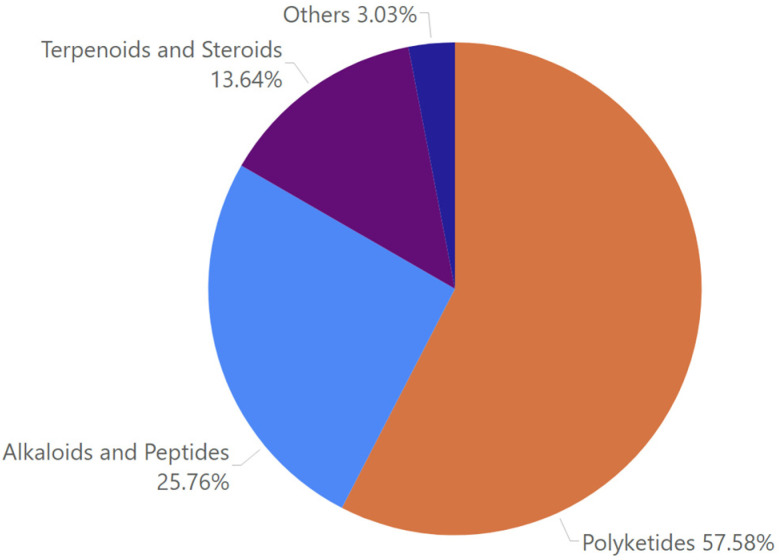
Antimycobacterial compounds from marine-derived fungi according to structure types.

The antimycobacterial MF discussed in this review consist of polyketides (57.58%), alkaloids and peptides (25.76%), terpenoids and steroids (13.64%), and miscellaneous compounds (3.03%) (Figure 1). Polyketides are some of the most varied metabolites, with distinct bioactivities identified in the fungi, which are known to house an immense number of biosynthetic gene clusters coding for numerous secondary metabolites, including polyketide synthase [9,10]. 

**Figure 2:**
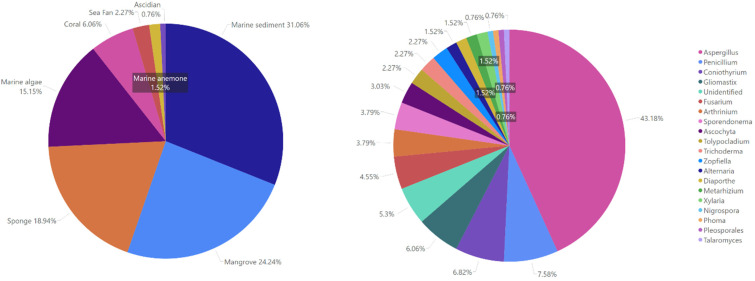
Marine sources of the fungi (**left**) and the antimycobacterial-producing genera (**right**).

Those antimycobacterials originate from 19 genera, including *Alternaria*, *Arthrinium*, *Ascochyta*, *Aspergillus*, *Coniothyrium*, *Diaporthe*, *Fusarium*, *Gliomastix*, *Metarhizium*, *Nigrospora*, *Penicillium*, *Phoma*, *Pleosporales*, *Sporendonema*, *Talaromyces*, *Tolypocladium*, *Trichoderma*, *Zopfiella*, and *Xylaria*, which were isolated from various marine sources such as marine sediment, mangrove, sponge, alga, coral, sea fan, anemone, and ascidian (Figure 2).

### 2.2. Novel Antimycobacterials from Marine-Derived Fungi

**Figure 3:**
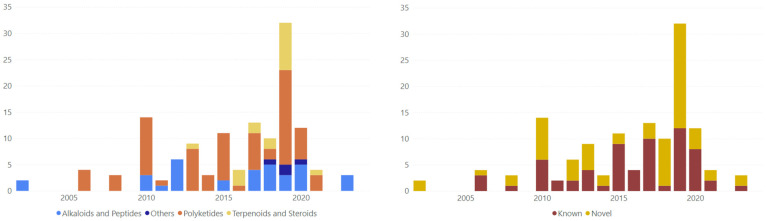
All marine-derived fungi metabolites by type/year (**left**) and novelty/year (**right**).

**Figure 4:**
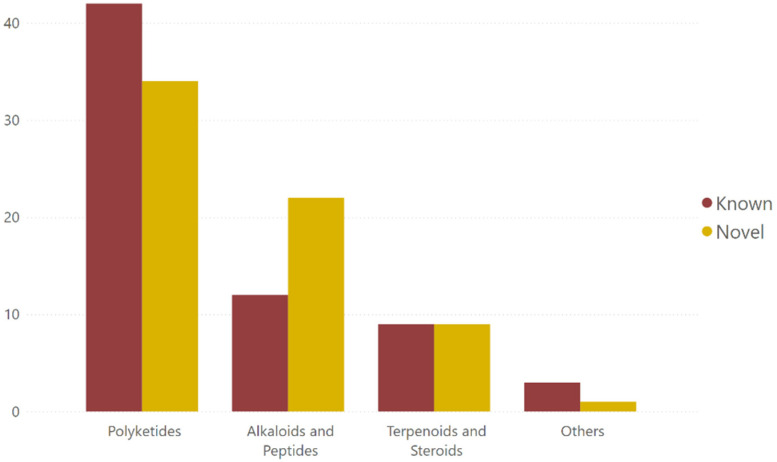
Antimycobacterial compounds based on the novelty as per structure types.

Between 2002 and 2023, roughly half of all marine-fungal antimycobacterial compounds reported each year were new (novel) structures (Figure 3). The majority of these novel compounds were found in 2019, which had the highest number of marine-fungal antimycobacterial compounds reported in a single year. Novel compounds were found more dominantly in the alkaloid and peptide groups compared to other compound types (Figure 4). Polyketides, being the largest group of MF antimycobacterials, are composed of more known compounds than novel ones (Figure 3). This is partly due to polyketides being one of the most abundant metabolites in fungi, leading to their more frequent discovery compared to other compound groups [11].

### 2.3. Antimycobacterial Assay

**Figure 5:**
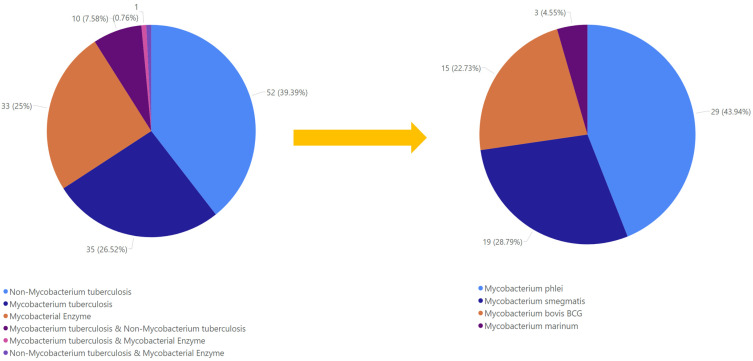
The *Mycobacterium* strains and enzymes used in the antimycobacterial assay.

We found that a total of 131 MF metabolites (out of 139) exhibited antimycobacterial activity, ranging from strong to weak activity (in the sections below, compounds with strong activity will be discussed in more detail; weak compounds will be described more briefly). The compounds were investigated against various *Mycobacterium* strains, such as *M. tuberculosis*, *M. smegmatis*, *M. bovis* BCG, *M. phlei*, and *M. marinum*. Another method that is commonly used to investigate antitubercular activity is the mycobacterial enzyme inhibitor assay. Here, we plot the activity against the *Mycobacterium* strain or specific enzyme (Figure 5).

Our review showed that 39.4% of compounds were tested only against non-*M. tuberculosis* species. Of those tests, *M. phlei* was the most common surrogate (accounting for 43.9% of the non-*M.tb* assays), followed by *M. smegmatis* (28.8%), *M. bovis* BCG (22.7%), and *M. marinum* (4.5%). In comparison, 26.3% of the compounds were tested against *M. tuberculosis* itself, and 25.0% were evaluated in enzymatic assays (e.g., against MptpB, MptpA, or PknG). A smaller fraction of compounds was tested in multiple model types: 7.6% against both *M. tuberculosis* and a surrogate species, 0.76% against both *M. tuberculosis* and a mycobacterial enzyme, and 0.76% against both a surrogate species and an enzyme. This suggests that surrogate models are highly valued as front-line, high-throughput surrogates that combine speed, safety, and cost-efficiency. 

### 2.4. Antimycobacterials Against Dormant Phenotypes

Tuberculosis infection is known to be highly influenced by the host’s immune status, indicating that the outcome post-invasion may differ from one individual to another. In immunocompetent individuals, infection can remain latent or become active, whereas immunocompromised hosts (e.g., HIV-positive patients) will most likely develop active disease [12,13]. Latent infection occurs when the host’s immune system contains the growth of the bacilli, preventing them from proliferating or causing tissue damage. This dormant (non-replicating) state is also thought to contribute to the need for prolonged therapy. Longer treatment duration, in turn, increase the risk of failure due to patient non-compliance, which can be triggered by severe and intolerable side effects of the drugs, the high cost of treatment, and the extended period during which patients remain contagious [12,14]. Vilcheze and co-workers suggested that most of the available antituberculosis therapies were only effective against metabolically active *Mycobacterium* and did not display the same potency against metabolically inactive clusters [15]. Hence, one of the solutions to this problem is to find active compounds that are effective not only against active *Mycobacterium* but also against the non-replicating ones [14]. 

**Figure 6:**
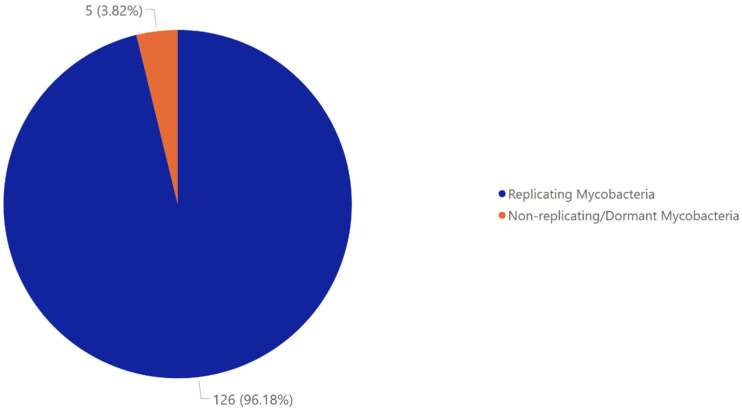
Antimycobacterial MF NPs against replicating and non-replicating *Mycobacterium*.

In this study, we specifically looked for marine-fungal compounds that had been tested against non-replicating (dormant) mycobacteria. Surprisingly, we found only five metabolites that had been evaluated for activity against dormant *Mycobacterium*, and those five did show activity (Figure 6). This highlights a major opportunity in which many marine-fungal metabolites active against replicating *M. tuberculosis* have yet to be tested against dormant bacteria.

### 2.5. Antimycobacterials from Marine-Derived Fungi

**Figure 7:**
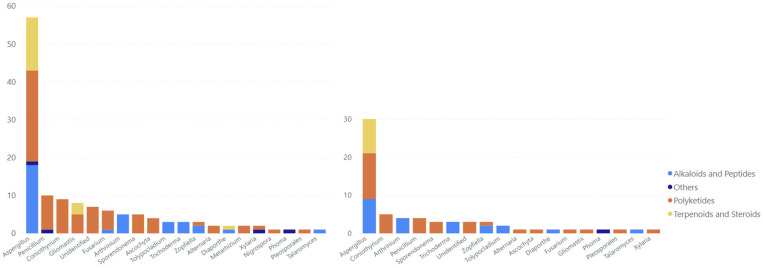
Compound distribution per producing genus: all compounds (**left**); novel compounds only (**right**).

The majority of the MF antimycobacterials were derived from *Aspergillus* (Figure 7), comprising polyketides as the most dominant compounds, followed by alkaloids and peptides and then steroids and terpenoids. The polyketides were also widely distributed in *Penicillium*, *Coniothyrium*, *Gliomastix*, *Fusarium*, *Sporendonema*, and *Ascochyta,* showing the abundance of polyketides in fungal metabolites. We found that the antimycobacterial compounds present in *Arthrinium*, *Tolypocladium*, *Trichoderma*, and *Talaromyces* were predominantly alkaloids and peptides. However, terpenoids and steroids with antimycobacterial properties were only identified in *Gliomastix* and *Diaporthe*, as well as *Aspergillus*.

There was a significant disparity in the number of compounds isolated from *Aspergillus* compared to other genera. *Aspergillus* yielded almost 60 antimycobacterial metabolites, whereas other genera produced fewer than 10 antimycobacterial compounds each. However, those compounds from the *Aspergillus* genus were derived from 20 *Aspergillus* species, yielding approximately 5–6 compounds per species, while other genera, such as *Fusarium* and *Penicillium,* consisted of five species each. Some of the genera only consisted of two species, such as *Gliomastix*, *Nigrospora*, and *Zopfiella,* and the rest only consisted of one species each. *Aspergillus* was found to be the most abundant genus in both marine and terrestrial environments and contributed most bioactive secondary metabolites, followed by *Penicillium*, making them the most potential sources for bioactive metabolite exploration, including novel compounds [16,17,18,19].

**Figure 8:**
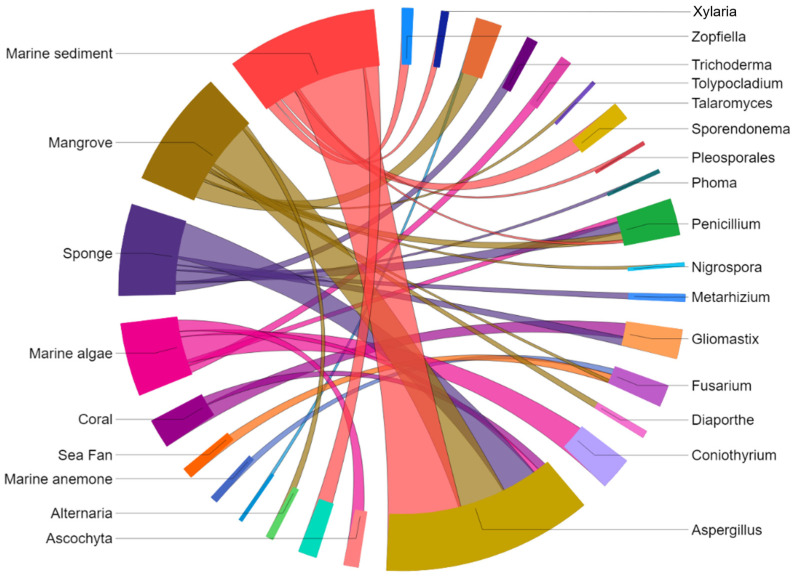
Fungal genera distribution in marine sources.

We attempted to plot the distribution of fungi from marine sources to determine the prevalence of fungal genera capable of producing antimycobacterial metabolites (Figure 8). Marine sediment contributed the greatest number of MF species with antimycobacterial metabolites, followed by mangroves, marine sponges, and marine algae. Fewer MF were obtained from sea fan, coral, marine anemone, and ascidian. *Aspergillus* producing antimycobacterial metabolites was mainly found in marine sediments, mangroves, and sponges. Some of the lesser-studied genera capable of producing novel antimycobacterial compounds were apparently endophytic fungi, such as *Coniothyrium* (derived from marine alga), *Trichoderma* (from marine sponge), *Talaromyces* (from mangrove), *Phoma* (from marine sponge), and *Tolypocladium* (from marine alga). Other promising but underexplored genera capable of producing novel compounds with antimycobacterial activity were derived from marine sediments, including *Arthrinium*, *Pleosporales*, *Sporendonema*, and *Zopfiella*.

In the following sub-sections, we describe in detail the activity of each compound, summarized in Table 1, Table 2, Table 3 and Table 4. To ease the categorization of the antimycobacterial activity, we recalculated any MIC or IC_50_ value from µg/mL into µM. We classified the antimycobacterial activity into strong, moderate, and weak activity based on the MIC or IC_50_ values. According to Cos and colleagues, anti-infectives from natural products can be categorized as “active” if the IC_50_ value is below 25 µM for pure compounds. Also, according to Elsebai, an inhibition zone of more than >15 mm from a 20 µg/disc of compound was considered to have considerable activity. Hence, we categorized compounds with IC_50_ ≤ 6.25 µM or inhibition zone > 15 mm as having strong activity, 6.25 µM < IC_50_ ≤ 25 µM or 10 mm < inhibition zone ≤ 15 mm as having moderate activity, and >25 µM or inhibition zone ≤ 10 mm as having weak activity. If the compound activity was stated by the MIC, then compounds with MIC ≤ 12.5 µM had strong activity, 12.5 µM < MIC ≤ 50 µM had moderate activity, and MIC > 50 µM had weak activity [20].

#### 2.5.1. Polyketides

Two polyketides, alterporriol S (**1**), a novel anthranoid dimer of the alterporriol type featuring a distinctive linkage between C-10 and C-2′, and a previously identified anthraquinone derivative, (+)-a*S*-alterporriol C (**2**), were obtained from the fungus *Alternaria* sp. SK11, which was collected from the mangrove *Excoecaria agallocha* (Shankou, Guangxi Province, China). Compound **2** displayed strong inhibitory activity against *M. tuberculosis* protein tyrosine phosphatase B (MptpB), a critical virulence factor released by *M. tuberculosis* to circumvent the host’s immune response, with an IC_50_ value of 8.70 µM. Meanwhile, compound **1** showed weak inhibitory activity against MptpB, with an IC_50_ value of 64.70 µM [21]. Compound **2** (IC_50_ 8.7 µM) adopted a classical C-10–C-10′ biaryl linkage that locked the two anthraquinone halves into an almost coplanar atropoisomeric scaffold compared with the twisted C-10–C-2′ link in compound **1** (IC_50_ 64.7 µM). The rigidity of compound **2** likely lowered the entropic penalty of binding and enlarged the contiguous π-surface, allowing stronger π-stacking with the hydrophobic P-loop clamp of MptpB and producing a seven-fold potency gain. The coplanar geometry simultaneously positioned an extra quinone carbonyl and a C-11 methoxy-ester to project toward the Lys164/Arg166 oxyanion region adjacent to the catalytic Asp165, creating a bidentate hydrogen-bond/phosphate-mimic network that compound **1**, with its twisted axis, could not achieve efficiently [21,22,23]. 

**Figure 9:**
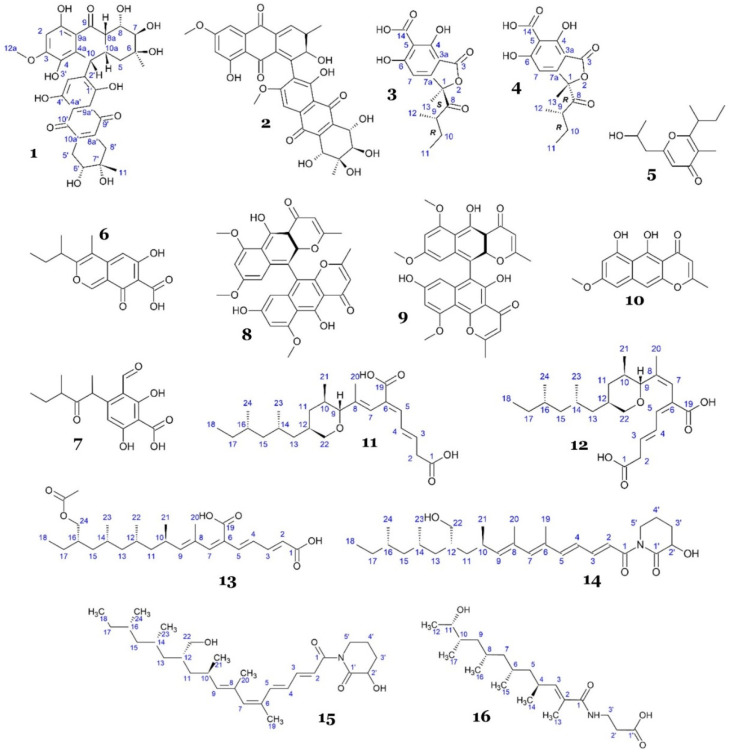
Chemical structures of compounds **1**–**16**.

An investigation of the marine alga-derived fungus *Ascochyta salicorniae* isolated from the North Sea, Germany, led to the isolation of new epimeric compounds (ascolactones A (**3**) and B (**4**)), along with other known compounds (hyalopyrone (**5**), ascochitine (**6**), and ascochital (**7**)) [24]. All compounds were tested against various phosphatases, including MptpB. However, only compound **6** exhibited moderate inhibition activity, with an IC_50_ of 11.5 µM, while compounds **4**, **5**, and **7** exhibited weak activities, with IC_50_ values of 95, 87.8, and 61.2 µM, respectively. Compound **3** showed no inhibitory activity. This suggested that the configuration at C-1 of compounds **3** and **4** (*R* or *S* configuration) was crucial for binding and affecting the activity (Figure 9). Within the 8-hydroxynaphthalenone series, the potency increased in the order hyalopyrone (**5**) < ascochital (**7**) < ascochitine (**6**), suggesting a positive contribution of the para-hydroxy-prenyl sidechain present only in **6** [24]. Compound **6** was the most potent member of this set (compounds **3**-**7**). Structurally, it was the only analogue that displayed an ortho-carboxylate flanked by two phenolic hydroxyls on a rigid chromone ring and retained two short, branched alkyl substituents. Together, these features likely match the polar Lys164/Arg166 oxyanion site and the adjacent Phe161/Tyr125 hydrophobic patch identified in multiple MptpB crystal structures, respectively [23,25]. Hyalopyrone (**5**) lacked carboxylate and a second phenol, hence giving it fewer hydrogen-bond donors/acceptors. Ascochital (**7**) preserved the dyad but lost one alkyl chain and planarity because of a saturated side arm. The ascolactones A (**3**) and B (**4**) introduced a spiro-lactone that distorted the phenol/acid alignment and, for epimer A (**3**), pointed the carbonyl lactone away from the catalytic Asp165, probably correlating with the sharp loss of activity. However, these geometric arguments remain hypotheses until corroborated by co-crystallography, docking with proper validation, or site-directed mutagenesis. 

Two novel dimeric naphtho-ɣ-pyrones, 8′-*O*-demethylnigerone (**8**) and 8′-*O*-demethylisonigerone (**9**), and a known analogue, rubrofusarin B (**10**) (Figure 9), were isolated from the fungal strain *Aspergillus carbonarius* WZ-4-11, obtained from marine sediment collected at Weizhou Island, Guangxi Province, China. Those compounds exhibited relatively moderate antimycobacterial activity against *M. tuberculosis* H37Rv, with MICs of 43 µM (**8** and **10**) and 21.5 µM (**9**) [26].

A chemical investigation of deep-sea-derived fungus *Aspergillus fischeri* FS452 led to the discovery of six new polypropionate derivatives with a unique long hydrophobic chain, fiscpropionates A–F (**11**–**16**) (Figure 9). Compounds **11**–**14** demonstrated strong to moderate inhibitory activity against MptpB, with IC_50_ values of 5.1, 12, 4.0, and 11 µM, respectively, while compounds **15** and **16** were inactive. Further kinetic experiments revealed that the inhibitory mechanism was noncompetitive. Interestingly, different activities between compounds **11**–**14** and **16** might have been derived from the different hydrophobicity and hydrophilicity of the opposing terminal functional groups of **11**–**14**. Compounds **11**–**14** possessed polar carboxyl and alkyl chains (C-12 to C-18) at the opposing terminal, while compound **16** was only substituted by the hydrophilic functional group at C-11. Furthermore, compound **14** was more active than **15**, which suggested the significance of the E configuration of the Δ^6^ double bond [27]. The potency in the fiscpropionate series likely tracked with the preservation of a tripartite pharmacophore: (1) an E-configured α,β-unsaturated carbonyl flanked by a β-OH; (2) a terminal carboxylate or imide; and (3) a linear, methyl-branched aliphatic chain [27]. Fiscpropionate C (**13**) (IC_50_ 4 µM) was most active likely because of its acyclic backbone, allowing for the optimal alignment of the enone/β-OH while keeping the required acid tail [23,25]. Fiscpropionate A (**11**) (IC_50_ 5.1 µM) was slightly less potent yet still benefited most likely from the intact conjugated β-hydroxy-ketone, whereas fiscpropionate B (**12**) (IC_50_ 12 µM) lost activity after the reduction of this motif, and fiscpropionate D (**14**) (IC_50_ 11 µM) suffered from the weaker, less acidic imide handle. We speculate that the inactive fiscpropionates E (**15**) and F (**16**) abolish key hydrogen-bonding vectors and over-alkylate the chain (or append a bulky aryl), further reducing binding by preventing proper insertion and electrostatic engagement, thus failing to inhibit the phosphatase. 

**Figure 10:**
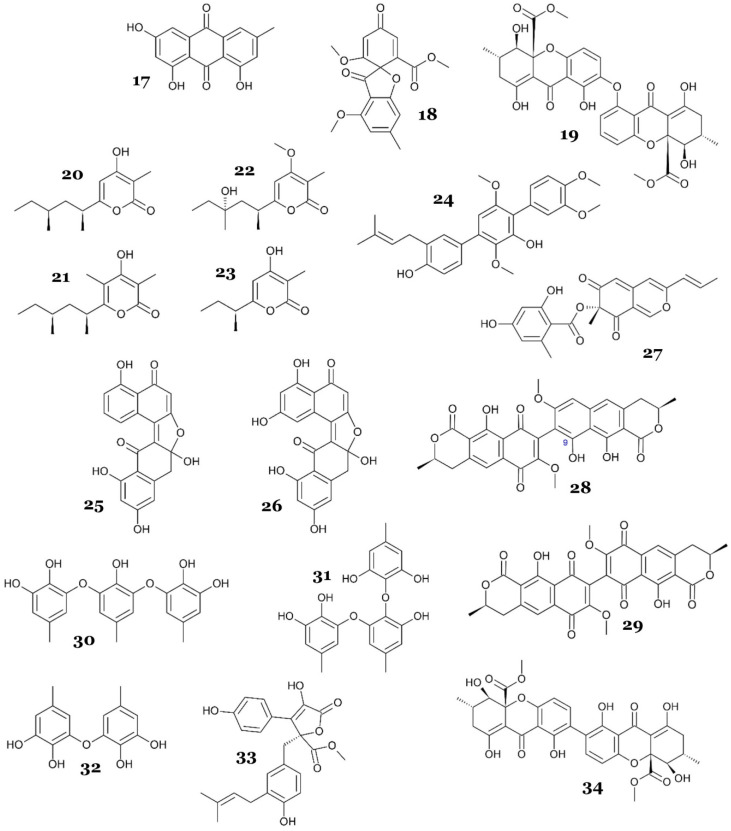
Chemical structures of compounds **17**–**34**.

Song and colleagues reported two compounds, emodin (**17**) and trypacidin (**18**) (Figure 10), discovered from *Aspergillus fumigatus* MF029 isolated from the marine sponge *Hymeniacidon perlevis*, obtained from the Bohai Sea, China. Both compounds demonstrated strong inhibition against *Mycobacterium bovis* BCG, with an MIC value of 1.25 µg/mL (4.6 and 3.63 µM, respectively) [28]. A separate study on **17** as an antimycobacterial revealed that its mechanism of action is targeting the G-quadruplex structure of mycobacterial genetic material. The molecule binds and thermally stabilizes G4 DNA motifs in the *mosR* (redox-stress regulator) and *ndhA* (NADH dehydrogenase) genes of *M. tuberculosis*, which suppresses their transcription and slows bacillary growth [29].

A new tetrahydroxanthone dimer, 5-*epi*-asperdichrome (**19**) (Figure 10), was discovered from *Aspergillus versicolor* HDN1009, isolated from soil around a mangrove area in Guangzhou, China. Compound **19** showed weak inhibitory activity against *Mycobacterium phlei*, with an MIC of 200 µM [30].

Solid rice fermentation of sponge-derived *Aspergillus niger* LS24 led to the discovery of three novel and one known 4-hydroxy-α-pyrones, nipyrones A–C (**20**–**22**) and germicidin C (**23**) (Figure 10), respectively. All compounds demonstrated weak antimycobacterial activity against *M. tuberculosis*, with MICs for **20**, **21**, and **23** of 128 µg/mL (570.66 µM, 537.093 µM, and 702.486 µM, respectively) and **22** of 64 µg/mL (251.652 µM) [31].

Prenylterphenyllin J (**24**) (Figure 10), a prenylated *p-terphenyl* isolated from the mangrove endophytic fungus *Aspergillus candidus* LDJ-5, showed weak antimycobacterial activity against *M. phlei*, with an IC_50_ of 45 µg/mL (99.88 µM). The fungus was obtained from the root of *Rhizophora apiculata* Blume collected from Sanya Bailu Park of Hainan Province, China [32].

A racemic of known dinaphthalenone derivatives, (±)-asperlone A (**25**), (±)-asperlone B (**26**), and (–)-mitorubrin (**27**) (Figure 10), were isolated from the mangrove-derived fungus *Aspergillus* sp. 16-5c. The fungal strain was obtained from leaves of the mangrove *Sonneratia apetala*, collected in Hainan Island, China. The compounds were tested against MptpB and demonstrated strong inhibitory activity, with IC_50_ values of 4.24, 4.32, and 3.99 µM, respectively [33]. Compounds **25**–**27** shared a rigid, nearly coplanar 1,4-diketone core flanked by phenolic or β-hydroxy groups. This carbonyl–phenol dyad is consistent with the dianionic pharmacophore required to engage the Lys164/Arg166 oxyanion pocket of MptpB, and the extended π-surface could plausibly interact with the hydrophobic Phe161–Tyr125 wall characteristic of the enzyme’s unusually wide active site [23,34,35]. All three compounds inhibited MptpB in the low-micromolar range, while peripheral variations such as a prenyl side chain (**27**) or methoxy substitution (**25**,**26**) appeared to fine-tune lipophilicity rather than alter the core binding motif [33].

Kamiya and colleagues re-discovered viomellein (**28**) and xanthomegnin (**29**) (Figure 10) from a culture of the *Aspergillus* sp. isolated from marine sponge obtained from Sabang Island, Indonesia [36]. The compounds exhibited antimycobacterial activity against *M. smegmatis* and *M. bovis* BCG in both active and dormant states. Dormant *Mycobacterium* is a state where the cells are metabolically inactive; it has slower replication and phenotypically increased resistance to current antituberculosis treatments [37]. Compound **28** demonstrated stronger activity against *M. bovis* BCG (with MICs of 6.25 µg/mL (11.15 µM) for aerobic and 1.56 µg/mL (2.78 µM) for hypoxic conditions) than against *M. smegmatis* (with MICs of 25 µg/mL (44.6 µM) for aerobic and 50 µg/mL (89.20 µM) for hypoxic conditions), while compound **29** showed the opposite, with an MIC against *M. smegmatis* for both aerobic and hypoxic conditions of 12.5 µg/mL (21.76 µM) and MICs against *M. bovis* BCG for aerobic conditions of 25 µg/mL (43.52 µM) and hypoxic conditions of 50 µg/mL (87.03 µM). The authors suggested that the different tendencies in the antimycobacterial activity from **28** and **29** against the tested *Mycobacterium* strains may derive from the hydroxyl group at C-9, which makes **28** more active against *M. bovis* BCG than *M. smegmatis*, compared with **29**. Interestingly, compound **28** also exhibited more potent activity against dormant *M. bovis* BCG compared with the control, isoniazid (MIC > 100 µg/mL) [36]. 

Two new tris-pyrogallol ethers, sydowiol A (**30**) and C (**31**) (Figure 10), and the known bis-pyrogallol ether, violaceol I (**32**), were discovered from the marine-derived fungus *Aspergillus sydowii* MF357, which was isolated from marine sediment in the East China Sea. Compounds **30** and **31** showed weak inhibitory activity against MptpA, another virulence factor secreted by *M. tuberculosis* that is responsible for tuberculosis pathogenicity by inhibiting phagosome–lysosome fusion [38], with IC_50_ values of 14 µg/mL (36.42 µM) and 24 µg/mL (62.44 µM), respectively, while **32** was inactive. Interestingly, an in vitro assay against *M. tuberculosis* H37Rv (in the same study) showed that compounds **30** and **31** were inactive, while compound **32** was weakly active, with an MIC of 25 µg/mL (95.33 µM). These results suggest that compounds **30** and **31** do not directly inhibit mycobacterial cells but rather neutralize the effect of MptpA, leading to enhanced host immune response and reduced viable bacteria. Although **32** may not demonstrate any alteration in the survival of mycobacterial cells post-infection of the macrophage, instead, it can directly inhibit mycobacterial cell growth [39]. The inhibition of MptpA likely improves when the scaffold can present three coplanar pyrogallol phenolates. Sydowiol A (**30**), with para/para ether bridges, retained full coplanarity and inhibited the phosphatase at IC_50_ = 14 µg/mL (36 µM). Shifting one bridge to an ortho position in sydowiol C (**31**) twisted the central ring, misaligned one phenolate, and reduced activity to IC_50_ = 24 µg/mL (62 µM), while both compounds showed only weak whole-cell activity (MIC > 50 µg/mL) against *M. bovis* BCG and *M. tuberculosis* H37Rv [39]. Violaceol I (**32**) contained only two pyrogallol units and was inactive against MptpA but displayed modest antibacterial activity (MIC = 25 µg/mL) that was attributed to non-specific oxidative stress. Although a co-crystal was lacking, molecular models based on the MptpA NMR structure (PDB 2LUO) suggested that three well-oriented phenolates were needed to satisfy the Lys21/Arg24 oxyanion site, whereas compounds offering only two donors formed sub-optimal contacts [40]. Hence, we speculate that the enzymatic potency correlated with the ability to deliver a coplanar, tri-dentate phenolate array, whereas whole-cell efficacy was governed by size and polarity rather than precise active-site binding.

A known compound, butyrolactone I (**33**) (Figure 10), was re-discovered from *Aspergillus terreus* SCSIO 41008, a marine-derived fungal isolated from sponge *Callyspongia* sp. obtained from Xuwen County, Guangdong, China. The compound exhibited strong, non-competitive inhibitory activity against MptpB, with an IC_50_ value of 5.11 µM [41]. Butyrolactone I (**33**) possessed a rigid, nearly coplanar γ-butyrolactone–aryl core bearing two conjugated carbonyls and flanking resorcinol OH groups. This carbonyl–phenol dyad is consistent with the dianionic pharmacophore that engages the Lys164/Arg166 oxyanion pocket of MptpB, and its extended π-surface could plausibly interact with the Phe161–Tyr125 hydrophobic wall seen in the crystal structure (PDB 2OZ5) [23,35]. The fused lactone rigidifies the aromatic array, lowering the entropic cost of binding, while the prenyl side chain may occupy part of the shallow lipophilic groove adjacent to Leu199. Together, these features rationalize the low-micromolar potency of butyrolactone I (IC_50_ = 5.11 µM) and mirror the design logic observed for other planar, phenolate-rich inhibitors such as (±)-asperlones (**25**–**26**) and (+)-a*S*-alterporriol C (**2**) [23].

A chemical investigation of the fermentation of marine sponge-derived *Aspergillus* sp. SCSIO XWS03F03 on a solid rice medium led to the isolation of secalonic acid D (**34**) (Figure 10). The bioactivity assay of **34** revealed that it demonstrated strong antimycobacterial activity against *M. tuberculosis*, with an IC_50_ of 1.26 µM [42].

**Figure 11:**
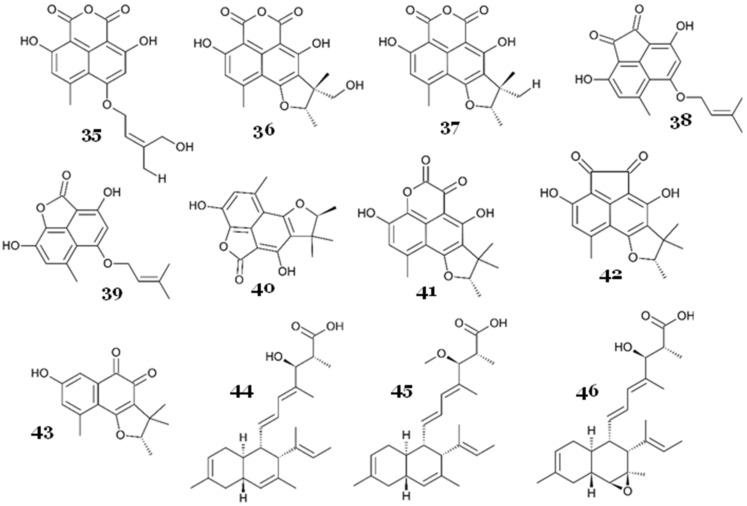
Chemical structures of compounds **35**–**46**.

Elsebai and colleagues reported nine compounds with antimycobacterial activity from *Coniothyrium cereale*, isolated from the marine alga *Enteromorpha* sp. collected from Fehmarn, the Baltic Sea. Five compounds were new phenalenone derivatives—(*Z*)-coniosclerodinol (**35**), (15*S*, 17*S*)-(–)-sclerodinol (**36**), conioscleroderolide (**37**), coniosclerodione (**38**), and coniolactone (**39**)—while the rest were known compounds, namely, (–)-7,8-dihydro-3,6-dihydroxy-1,7,7,8-tetramethyl-5*H*-furo-[2’,3’:5,6]naphtho[1,8-bc]furan-5-one (**40**), (–)-scleroderolide (**41**), (–)-sclerodione (**42**), and (–)-trypethelone (**43**) (Figure 11). Those compounds exhibited antimycobacterial activity against *M. phlei*, with the inhibition zone in the range of 10 – 22 mm at a concentration of 20 µg/disc [43,44]. 

New fusarielins, fusarielin M (**44**) and N (**45**), along with a known fusarielin, fusarielin G (**46**), were isolated from the marine-derived fungus *Fusarium graminearum* SYSU-MS5127, isolated from sea anemone obtained from Laishizhou Island, Shenzhen City, Guangdong Province, China. Those compounds were tested against MptpB, MptpA, and human protein tyrosine phosphatase 1B (PTP1B). Compound **44** demonstrated strong MptpB inhibitory activity, with an IC_50_ value of 1.05 µM. It also inhibited MptpA (IC_50_ = 23.78 µM) and PTP1B (IC _50_ = 15.74 µM), which indicated high specificity for MptpB over MptpA and PTP1B. Compound **45** showed no measurable inhibition against MptpB (IC _50_ > 40 µM), which suggested that the hydroxyl group at C-3 fusarielin (Figure 11) possessed essential activity as an MptpB inhibitor. Moreover, compound **46** showed less potency as an MptpB inhibitor (IC_50_ = 23.75 µM) compared with **44**, which indicated that MptpB inhibition was hampered by the decalin’s moiety epoxy bond group (Figure 11). An investigation of the cellular activity of **44** revealed that **44** restored the MAPK signaling pathway, which was affected by MptpB. Furthermore, an increased concentration of **44** significantly reduced the mycobacterial growth inside the macrophage without altering macrophage viability, which excluded the possibility of cytotoxicity effects of high concentrations of **44** against mycobacterial cells. An in silico study showed that **44** resided inside the MptpB active site, connected by a hydrogen bond from the carboxylate group of **44** with the side chain of Asp165, a residue in the phosphate-binding loop (P-loop). Asp165 was reported as a unique feature of MptpB [45], which may explain the selectivity of **44** as an MptpB inhibitor [46]. Docking into the 2OZ5 crystal structure predicted that its free C-3 β-OH could hydrogen-bond to the catalytic Asp165, while the nearly planar decalin–polyene backbone may extend along the Phe161/Tyr125 hydrophobic wall [23]. O-methylation of the hydroxyl (**45**) or epoxidation of the decalin core (**46**) reduced activity, supporting a working model in which (i) an unhindered C-3 phenolic donor, (ii) a rigid conjugated polyene that fits the shallow lipophilic groove, and (iii) limited steric bulk around the phenol are key for high affinity. Direct co-crystal or kinetic data with MptpA and human PTPs are still lacking, so the exact binding pose remains to be confirmed.

**Figure 12:**
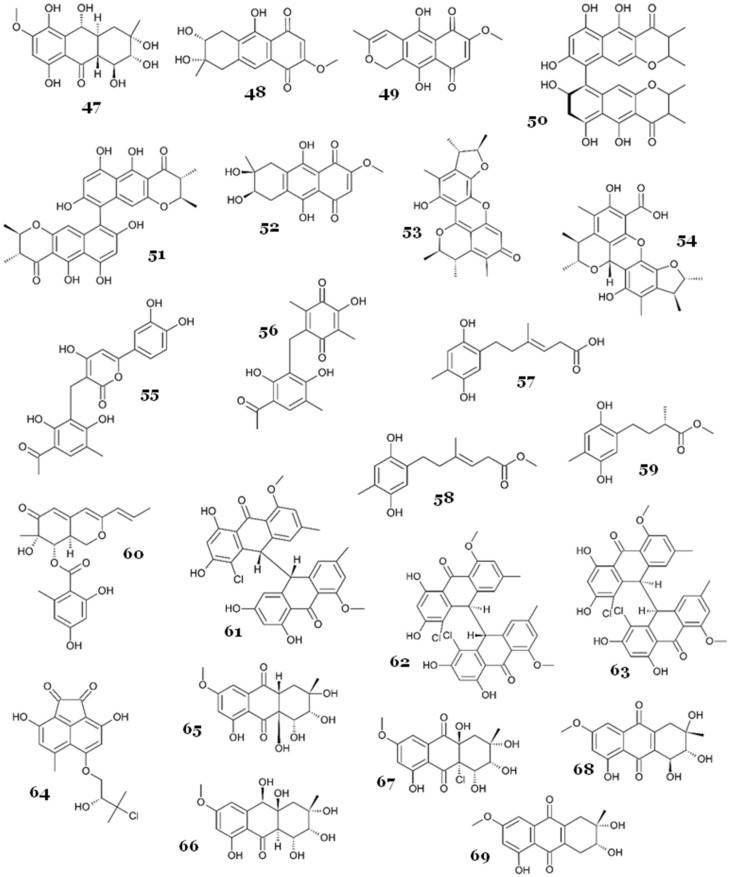
Chemical structures of compounds **47**–**69**.

Three compounds, 9α-hydroxyhalorosellinia (**47**), nigrosporin B (**48**), and anhydrofusarubin (**49**) (Figure 12), were re-discovered from marine-derived *Fusarium* spp. PSU-F14 and PSU-F135, isolated from gorgonian sea fan (*Annella* sp.) collected near Koh Hin Ran Pet, Suratthani Province, Thailand. Those compounds exhibited moderate antimycobacterial activity against *M. tuberculosis* H37Ra, with MIC values of 39, 41, and 47 µM, respectively [47].

An investigation of the extract of *Metarhizium anisopliae* mxh-99, isolated from marine sponge from Naozhou Island, China, led to the isolation of isochaetochromin B_2_ (**50**) and ustilaginoidin D (**51**) (Figure 12). Both compounds demonstrated weak antimycobacterial activity against *M. phlei*, with an MIC value of 50 µg/mL (91.15 and 91.49 µM, respectively) [48].

4-deoxybostrycin (**52**) and its deoxy-derivative, **48**, were isolated from the endophytic fungus *Nigrospora* sp., which was obtained from the decayed wood of mangrove *Kandelia candel* (L.) Druce, collected from Mai Po, Hong Kong. The compounds were tested against various *M.* strains, both sensitive and resistant to antituberculosis agents (drug-resistant). Compound **52** was moderately active against *M. bovis* BCG, *M. tuberculosis* H37Rv, the clinical MDR *M. tuberculosis* strain K2903531 (resistant to SM, INH, RF, and ETH), the clinical MDR *M. tuberculosis* strain 0907961 (resistant to SM and ETH), the clinical drug-resistant *M. tuberculosis* strain K0903557 (resistant to INH), and clinical drug-sensitive *M. tuberculosis*, with MIC values of 39, 15, <5, 10, 30, and 10 µg/mL, respectively (121.76, 46.83, 15.61, 31.22, 93.67, and 31.22 µM, respectively), while **48** was also moderately active against *M. bovis* BCG, *M. tuberculosis* H37Rv, the clinical MDR *M. tuberculosis* strain K2903531 (resistant to SM, INH, RF, and ETH), the clinical MDR *M. tuberculosis* strain 0907961 (resistant to SM and ETH), and the clinical drug-resistant *M. tuberculosis* strain K0903557 (resistant to INH), with MIC values of 15, 20, 30, 20, and 30 µg/mL, respectively (49.30, 65.73, 98.59, 65.73, and 98.59 µM, respectively) [49]. 

An endophytic fungus, *Penicillium citrinum* WK-P9, isolated from the marine sponge *Suberea* sp., produced two known citrinin derivatives, penicitrinone A (**53**) and penicitrinol J (**54**) (Figure 12). Compounds **53** and **54** exhibited weak antimycobacterial properties against *M. smegmatis* ATCC607, with an MIC value of 32 µg/mL (84.11 and 75.04 µM, respectively) [50]. 

Two new peniphenones, peniphenone B (**55**) and C (**56**) (Figure 12), with MptpB inhibitory activity were discovered from *Penicillium dipodomyicola* HN4-3A. The fungal strain was isolated from the stem of *Acanthus ilicifolius* obtained from the South China Sea, Hainan Province, China. Compounds **55** and **56** exhibited strong inhibitory properties against MptpB in vitro, with IC_50_ values of 0.16 and 1.37 µM, respectively [51]. Compound **55** was 9-fold more potent than its oxidized analogue **56**. A plausible explanation is that **55** retains three phenolic OH groups (one on the lower resorcinol ring and an ortho-dihydroxyl (catechol) pair on the upper ring) and an ether oxygen, in which these four hetero-atoms provide multiple hydrogen-bond donors/acceptors that could engage the polar residues lining the MptpB active site, while the fully aromatic, conjugated scaffold supplies an extended π-surface for hydrophobic or π-stacking contacts with residues such as Tyr125 and Phe161 that flank the shallow binding cleft [34,35]. In compound **56**, oxidation of the upper catechol to a para-benzoquinone replaced two hydroxyl donors with carbonyl groups and left only a single phenol available for deprotonation; hence, concomitant electron withdrawal was expected to lower the basicity of the remaining OH and central benzophenone carbonyl, cumulatively weakening potential hydrogen-bond and electrostatic interactions [51]. Because the overall backbone and substitution pattern remained similar, the loss of potency in compound **56** can be reasonably attributed to the reduced number and strength of polar contacts rather than to major conformational effects.

An Antarctica-associated fungi, *Penicillium* sp. HDN151272, isolated from unidentified sponge, produced three new polyketides, ketidocillinones A–C (**57**–**59**). All compounds were tested against *M. phlei*, with only **58** and **59** exhibiting antimycobacterial activity, with MIC values of 3.13 and 6.25 µg/mL, respectively (11.84 and 26.23 µM, respectively). Compound **57** did not show inhibition activity against *M. phlei*, which indicated the importance of the methoxy group of compounds **58** and **59** (Figure 12) for the antimycobacterial activity [52]. 

A mangrove-associated fungus, *Penicillium pinophilum* SCAU037, produced a known hydrogenated azaphilone, Sch725680 (**60**), which exhibited moderate antimycobacterial activity against *M. smegmatis*, with an MIC value of 23.5 µM [53]. 

Further chemical investigation of a marine alga-derived *Penicillium roseopurpureum* KP1-135C extract led to the isolation of three halogenated bianthrones, neobulgarones D–F (**61**–**63**), and their antibacterial activities were investigated. The study reported that only the *cis* isomers, compounds **61** and **63** (Figure 12), exhibited weak antimycobacterial activity against *M. tuberculosis* H37Ra ATCC 25177, with IC_50_ values of 46.1 and 31.1 µM, respectively. The *trans* isomer, **62**, however, did not show antimycobacterial activity but exhibited moderate inhibition against methicillin-resistant *Staphylococcus aureus*, which indicated that the *cis-trans* configuration of neobulgarones may affect the selectivity of antibacterial–antimycobacterial activity [54]. 

A marine sediment-derived *Pleosporales* sp. HDN1811400 produced a new phenalenone derivative, peniciphenalenin G (**64**), and two known analogues, **36** and **38**, which exhibited antimycobacterial activity against *M. phlei*, with MIC values of 50, 25, and 25 µM, respectively [55]. 

New anthraquinone derivatives, auxarthrols D (**65**), F (**66**), and G (**67**), along with two known analogues, 4-dehydroxyaltersolanol A (**68**) and altersolanol B (**69**) (Figure 12), were isolated from *Sporendonema casei* HDN16-802, which was obtained from marine sediment from Liaoning Province, China. All compounds exhibited antimycobacterial activity against *M. phlei*, with MIC values in the range of 25–200 µM. However, only **69** exhibited inhibitory activity against *M. tuberculosis*, with an MIC value of 20 µM [56]. 

**Figure 13:**
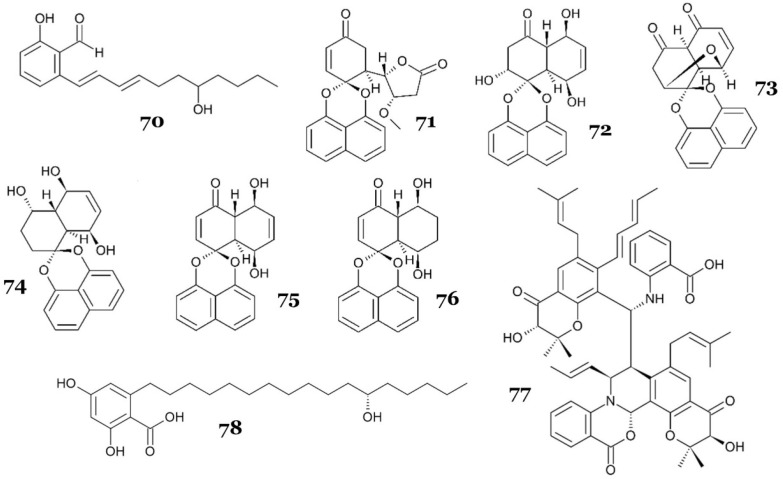
Chemical structures of compounds **70**–**78**.

Marine-derived fungus *Zopfiella marina* produced a new salicylaldehyde derivative, 2-hydroxy-6-((1E,3E)-7-hydroxyundeca-1,3dienyl)benzaldehyde (**70**) (Figure 13), with weak antimycobacterial activity against *M. tuberculosis* H37Ra, with an MIC value of 25 µg/mL (86.69 µM) [57]. 

Bunyapaiboonsri and colleagues reported an unidentified mangrove-associated fungus BCC 25093, which produced two new palmarumycins, palmarumycins P1 (**71**) and P3 (**72**) (Figure 13), along with two known palmarumycins, palmarumycins CP3 (**73**) and CR1 (**74**), and two known decaspirones, decaspirones A (**75**) and C (**76**), with potential antimycobacterial activity. The strongest activity was shown by **71** and **75**, with an IC_50_ value of 1.56 µg/mL (4.23 and 4.64 µM, respectively), followed by **73** and **76** (IC_50_ = 3.13 µg/mL or 9.36 and 9.25 µM, respectively) and **72** and **74** (IC_50_ = 12.5 µg/mL or 35.28 and 36.51 µM, respectively) [58]. 

Another unidentified, novel, marine-derived fungus strain 110162 in the Eurotiomycetes class was reported to produce a racemic of prenylated polyketide dimer, oxazinin A (**77**), with a pentacyclic structure formed with an unusual combination of benzoxazine, isoquinoline, and a pyran ring (Figure 13). Compound **77** also exhibited a strong antimycobacterial activity against *M. tuberculosis*, with an IC_50_ value of 2.9 µM [59]. 

Penixylarin C (**78**), a novel alkyl aromatic compound (Figure 13), was isolated from a mangrove-associated fungus *Xylaria* sp. HDN13-249. It exhibited a strong antimycobacterial activity against *M. phlei*, with an MIC value of 6.25 µM [60]. 

**Table 1:** Polyketide NPs with antimycobacterial activity from MF.

No.	Metabolites [Novelties at the Time of Isolation]	Producing Strains	Marine Sources	Fermentation Media and Method	Tested Against Mycobacterium Strain/Mycobacterial Enzyme	Potency	Mechanism of Action	Ref.
**1**	alterporriol S [N] ^a^	*Alternaria* sp. SK1	Root of mangrove *Excoecaria agallocha*	Potato glucose liquid medium, static condition, 26 °C, 4 weeks	Mycobacterial Enzyme MptpB	IC_50_ = 64.7 µM	Inhibit virulence factor MptpB	[21]
**2**	(+)-a*S*-alterporriol C [K] ^b^					IC_50_ = 8.7 µM		
**3**	ascolactone A [N]	*Ascochyta salicorniae*	Marine alga *Ulva* sp.	Solid medium (biomalt extract 2%, agar 1.5%, ASW * 80%), static condition, room temp., 52 days	Mycobacterial Enzyme MptpB	NA **	-	[24]
**4**	ascolactone B [N]					IC_50_ = 95 µM	Inhibit virulence factor MptpB	
**5**	hyalopyrone [K]					IC_50_ = 87.8 µM		
**6**	ascochitine [K]					IC_50_ = 11.5 µM		
**7**	ascochital [K]					IC_50_ = 61.2 µM		
**8**	8′-*O*-demethylnigerone [N]	*Aspergillus carbonarius* WZ-4-11	Marine sediment	Liquid medium (glucose 2%, peptone 0.5%, malt extract 0.3%, yeast extract 0.3%, sea water pH 7.0), static condition, 24 °C, 30 days	*M. tuberculosis* H37Rv	IC_50_ = 43 µM	NR ***	[26]
**9**	8′-*O*-demethylisonigerone [N]					IC_50_ = 21.5 µM		
**10**	rubrofusarin B [K]					IC_50_ = 43 µM		
**11**	fiscpropionate A [N]	*Aspergillus fisheri* FS452	Marine sediment	Solid medium (rice 250 g, 400 mL H_2_O, natural sea salt 0.3%), static condition, room temp., 30 days	Mycobacterial Enzyme MptpB	IC_50_ = 5.1 µM	Inhibit virulence factor MptpB	[27]
**12**	fiscpropionate B [N]					IC_50_ = 12 µM		
**13**	fiscpropionate C [N]					IC_50_ = 4 µM		
**14**	fiscpropionate D [N]					IC_50_ = 11 µM		
**15**	fiscpropionate E [N]					NA	-	
**16**	fiscpropionate F [N]					NA	-	
**17**	emodin [K]	*Aspergillus fumigatus* MF029	Marine sponge *H. perleve*	Solid medium (160 g rice, 200 mL H_2_O), static condition, 28 °C, 30 days	*M. bovis* BCG	IC_50_ = 1.25 µg/mL (4.6 µM)	Bind and thermally stabilize G4 DNA motifs in the *mosR* (redox-stress regulator) and *ndhA* (NADH dehydrogenase) genes of *M. tuberculosis*	[28,29]
**18**	trypacidin [K]					IC_50_ = 1.25 µg/mL (3.63 µM)	NR	
**19**	5-*epi*-asperdichrome [N]	*Aspergillus versicolor* HDN1009	Soil around mangrove	Liquid medium (maltose 2%, mannitol 2%, glucose 1%, monosodium glutamate 1%, MgSO_4_.7H_2_O 0.03%, KH_2_PO_4_ 0.05%, yeast extract 0.3%, corn steep liquor 0.1%, ASW), static condition, 28 °C, 14 days	*M. phlei*	MIC = 200 µM	NR	[30]
**20**	nipyrone A [N]	*Aspergillus niger* LS24	Marine sponge *Haliclona* sp.	Solid medium (100 g rice, 160 mL H_2_O), static condition, 28 °C, 30 days	*M. tuberculosis* H37Rv	MIC = 128 µg/mL (570.66 µM)	NR	[31]
**21**	nipyrone B [N]					MIC = 128 µg/mL (537.093 µM)		
**22**	nipyrone C [N]					MIC = 64 µg/mL (251.652 µM)		
**23**	germicidin C [K]					MIC = 128 µg/mL (702.486 µM)		
**24**	prenylterphenyllin J [K]	*Aspergillus candidus* LDJ-5	Root of mangrove *Rhizophora apiculata* Blume	Liquid medium (mannitol 2%, monosodium glutamate 1%, maltose 3%, yeast extract 0.3%, glucose 1%, corn steep liquor 0.1%, magnesium sulfate heptahydrate 0.03%, monopotassium phosphate 0.05%, H_2_O), static condition, 28 °C, 30 days	*M. phlei*	MIC = 45 µg/mL (99.88 µM)	NR	[32]
**25**	(±)-asperlone A [K]	*Aspergillus* sp. 16-5c	Leaves of mangrove *Sonneratia apetala*	Liquid medium (glucose 1.5%, sea salt 0.3% in potato infusion), static condition, 28 °C, 30 days	Mycobacterial Enzyme MptpB	IC_50_ = 4.24 µM	Inhibit virulence factor MptpB	[33]
**26**	(±)-asperlone B [K]					IC_50_ = 4.32 µM		
**27**	(–)-mitorubrin [K]					IC_50_ = 3.99 µM		
**28**	viomellein [K]	*Aspergillus* sp. 02E28_2-2	Unidentified marine sponge	Solid medium (250 g unpolished rice, 500 ASW), static condition, 30 °C, 14 days	*M. smegmatis* mc^2^155	MIC aerobic = 25 µg/mL (44.6 µM); hypoxic = 50 µg/mL (89.20 µM)	NR	[36]
					*M. bovis* BCG	MIC aerobic = 6.25 µg/mL (11.15 µM); hypoxic = 1.56 µg/mL (2.78 µM)		
**29**	xanthomegnin [K]				*M. smegmatis* mc^2^155	MIC aerobic = 12.5 µg/mL (21.76 µM); hypoxic = 12.5 µg/mL (21.76 µM)		
					*M. bovis* BCG	MIC aerobic = 25 µg/mL (43.52 µM); hypoxic = 50 µg/mL (87.03 µM)		
**30**	sydowiol A [N]	*Aspergillus sydowii* MF357	Marine sediment	Solid medium (130 g rice, 80 mL ASW), static condition, 25 °C, 20 days	Mycobacterial Enzyme MptpA	IC_50_ = 14 µg/mL (36.42 µM)	Inhibit virulence factor MptpA	[39]
					*M. bovis* BCG	NA	-	
					*M. tuberculosis* H37Rv	NA	-	
**31**	sydowiol C [N]				Mycobacterial Enzyme MptpA	IC_50_ = 24 µg/mL (62.44 µM)	Inhibit virulence factor MptpA	
					*M. bovis* BCG	MIC = 100 µg/mL (260.16 µM)	NR	
					*M. tuberculosis* H37Rv	NA	-	
**32**	violaceol I [K]				Mycobacterial Enzyme MptpA	NA	-	
					*M. bovis* BCG	NA	-	
					*M. tuberculosis* H37Rv	MIC = 25 µg/mL (95.33 µM)	NR	
**33**	butyrolactone I [K]	*Aspergillus terreus* SCSIO 41008	Marine sponge *Callyspongia* sp.	Liquid medium (potato 20%, peptone 0.5%, mannitol 2%, maltose 2%, glucose 2%, monosodium glutamate 0.5%, yeast extract 0.3%, sea salt 2%) in fermenter (28 °C, 135 rpm, 12 L/min aseptic air, 3 Mpa for 7 days)	Mycobacterial Enzyme MptpB	IC_50_ = 5.11 µM	Inhibit virulence factor MptpB	[41]
**34**	secalonic acid D [K]	*Aspergillus* sp. SCSIO XWS03F03	Unidentified marine sponge	Solid medium (200 g rice, 2 g sea salt, 200 mL H_2_O, supplemented with NaCl 1%), static condition, 25 °C, 45 days	*M. tuberculosis*	IC_50_ = 1.26 µM	NR	[42]
**35**	(*Z*)-coniosclerodinol [N]	*Coniothyrium cereale*	Marine alga *Enteromorpha* sp.	Solid BMS medium, static condition, room temp., 40 days	*M. phlei*	ZI = 16 mm	NR	[43,44]
**36**	(15*S*, 17*S*)-(–)-sclerodinol [N]					ZI = 20 mm		
**37**	conioscleroderolide [N]					ZI = 10 mm		
**38**	coniosclerodione [N]					ZI = 12 mm		
**39**	coniolactone [N]					ZI = 22 mm		
**40**	(–)-7,8-dihydro-3,6-dihydroxy-1,7,7,8-tetramethyl-5*H*-furo-[2’,3’:5,6]naphtho[1,8-bc]furan-5-one [K]					ZI = 12 mm		
**41**	(–)-scleroderolide [K]					ZI = 14 mm		
**42**	(–)-sclerodione [K]					ZI = 10 mm		
**43**	(–)-trypethelone [K]					ZI = 18 mm		
**44**	fusarielin M [N]				Mycobacterial Enzyme MptpB	IC_50_ = 1.05 µM	Inhibit virulence factor MptpB	[46]
					Mycobacterial Enzyme MptpA	IC_50_ = 23.78 µM	Inhibit virulence factor MptpA	
**45**	fusarielin N [N]				Mycobacterial Enzyme MptpB	NA	-	
					Mycobacterial Enzyme MptpA	NA	-	
**46**	fusarielin G [K]				Mycobacterial Enzyme MptpB	IC_50_ = 23.75 µM	Inhibit virulence factor MptpB	
					Mycobacterial Enzyme MptpA	NA	-	
**47**	9α-hydroxyhalorosellinia [K]	*Fusarium* spp. PSU-F15	Marine gorgonian sea fan *Annella* sp.	Liquid medium (potato dextrose broth), static condition, room temp., 28 days	*M. tuberculosis* H37Ra	MIC = 39 µM	NR	[47,49]
					*M. bovis* BCG	NA		
					*M. tuberculosis* H37Rv	NA		
					Clinical MDR *M. tuberculosis* strain (K2903531, resistant to SM, INH, RFP, and EMB)	NA		
					Clinical MDR *M. tuberculosis* strain (0907961, resistant to SM and EMB)	NA		
					Clinical drug-resistant *M. tuberculosis* strain (K0903557, resistant to INH)	NA		
**48**	nigrosporin B [K]				*M. tuberculosis* H37Ra	MIC = 41 µM		
					*M. bovis* BCG	MIC = 15 µg/mL (49.30 µM)		
					*M. tuberculosis* H37Rv	MIC = 20 (65.73 µM) µg/mL		
					Clinical MDR *M. tuberculosis* strain (K2903531, resistant to SM, INH, RFP, and EMB)	MIC = 30 (98.59 µM) µg/mL		
					Clinical MDR *M. tuberculosis* strain (0907961, resistant to SM and EMB)	MIC = 20 (65.73 µM) µg/mL		
					Clinical drug-resistant *M. tuberculosis* strain (K0903557, resistant to INH)	MIC = 30 (98.59 µM) µg/mL		
**49**	anhydrofusarubin [K]				*M. tuberculosis* H37Ra	MIC = 87 µM		
					*M. bovis* BCG	NA		
					*M. tuberculosis* H37Rv	NA		
					Clinical MDR *M. tuberculosis* strain (K2903531, resistant to SM, INH, RFP, and EMB)	NA		
					Clinical MDR *M. tuberculosis* strain (0907961, resistant to SM and EMB)	NA		
					Clinical drug-resistant *M. tuberculosis* strain (K0903557, resistant to INH)	NA		
**50**	isochaetochromin B_2_ [K]	*Metarhizium anisopliae* mxh-99	Unidentified marine sponge	Liquid medium (mannitol 2%, maltose 2%, glucose 1%, monosodium glutamate 1%, KH_2_PO_4_ 0.05%, MgSO_4_.7H_2_O 0.03%, yeast extract 0.3%, corn steep liquor 0.1%, ASW pH 6.5), agitated condition (165 rpm), 28 °C, 8 days	*M. phlei*	MIC = 50 µg/mL (91.15 µM)	NR	[48]
**51**	ustilaginoidin D [K]					MIC = 50 µg/mL (91.49 µM)		
**52**	4-deoxybostrycin [K]	*Nigrospora* sp.	Unidentified sea anemone	Fermentation from *Nigrospora* sp. was not explained in detail	*M. bovis* BCG	MIC = 39 µg/mL (121.76 µM)	NR	[49]
					*M. tuberculosis* H37Rv	MIC = 15 µg/mL (46.83 µM)		
					Clinical MDR *M. tuberculosis* strain K2903531 (resistant to SM, INH, RF, and ETH)	MIC = < 5 µg/mL (< 15.61 µM)		
					Clinical MDR *M. tuberculosis* strain 0907961 (resistant to SM and ETH)	MIC = 10 µg/mL (31.22 µM)		
					Clinical drug-resistant *M. tuberculosis* strain K0903557 (resistant to INH)	MIC = 30 µg/mL (93.67 µM)		
					Clinical drug-sensitive *M. tuberculosis*	MIC = 10 µg/mL (31.22 µM)		
**53**	penicitrinone A [K]	*Penicillium citrinum* WK-P9	Marine sponge *Suberea* sp.	Liquid medium (malt extract broth), static condition, 24 °C, 12 days	*M. smegmatis* ATCC 607	MIC = 32 µg/mL (84.11 µM)	NR	[50]
**54**	penicitrinol J [K]					MIC = 32 µg/mL (75.04 µM)		
**55**	peniphenone B [N]	*Penicillium dipodomyicola* HN4-3A	Stem of mangrove *Acanthus ilicifolius*	Liquid medium (20 g glucose, 2 g sea salt in 1 L of potato infusion), static condition, 25–30 °C, 30 days	Mycobacterial Enzyme MptpB	IC_50_ = 0.16 µM	Inhibit virulence factor MptpB	[51]
**56**	peniphenone C [N]					IC_50_ = 1.37 µM		
**57**	ketidocillinone A [N]	*Penicillium* sp. HDN151272	Unidentified marine sponge	Liquid medium ((glucose 1%, maltose 2%, mannitol 2%, monosodium glutamate 1%, KH_2_PO_4_ 0.05%, MgSO_4_.7H_2_O 0.03%, corn steep liquor 0.1%, yeast extract 0.3% in addition to natural sea water pH 6.5), agitated condition, 28 °C, 9 days	*M. phlei*	NA	NR	[52]
**58**	ketidocillinone B [N]					MIC = 3.13 µg/mL (11.84 µM)		
**59**	ketidocillinone C [N]					MIC = 6.25 µg/mL (26.23 µM)		
**60**	Sch725680 [K]	*Penicillium pinophilum* SCAU037	Roots of mangrove *Rhizophora stylosa*	Liquid medium (yeast extract 0.3%, malt extract 0.3%, peptone 0.5%, glucose 2%, sorbitol 2%, sea salt 3%), static condition, 28 °C, 30 days	*M. smegmatis* ATCC 607	23.5 µM	NR	[53]
**61**	neobulgarone D [K]	*Penicillium roseopurpureum* KP1-13	Brown alga *Petalonia fascia*	Liquid medium (malt extract broth 2%), agitated condition at 150 rpm, room temp., 14 days	*M. tuberculosis* H37Ra ATCC 25177	IC_50_ = 46.1 µM	NR	[54]
**62**	neobulgarone E [K]					NA		
**63**	neobulgarone F [K]					IC_50_ = 31.1 µM		
**64**	peniciphenalenin G [N]	*Pleosporales* sp. HDN1811400	Marine sediment	Liquid medium (yeast extract 0.3%, malt extract 0.3%, peptone 0.5%, glucose 2% dissolved in naturally collected seawater), static condition, 28 °C, 35 days	*M. phlei*	MIC = 50 µM	NR	[55]
**65**	auxarthrol D [N]	*Sporendonema casei* HDN16-802	Marine sediment	Solid medium (53 g oatmeal, 125 mL natural seawater), static condition, room temp., 30 days	*M. phlei*	MIC = 25 µM	NR	[56]
					*M. tuberculosis*	NA		
**66**	auxarthrol F [N]				*M. phlei*	MIC = 200 µM		
					*M. tuberculosis*	NA		
**67**	auxarthrol G [N]				*M. phlei*	MIC = 50 µM		
					*M. tuberculosis*	NA		
**68**	4-dehydroxyaltersolanol A [K]				*M. phlei*	MIC = 25 µM		
					*M. tuberculosis*	NA		
**69**	altersolanol B [K]				*M. phlei*	MIC = 25 µM		
					*M. tuberculosis*	MIC = 20 µM		
**70**	2-hydroxy-6-((1E,3E)-7-hydroxyundeca-1,3dienyl)benzaldehyde [N]	*Zopfiella marina*	Marine sediment	Liquid medium (glucose 4%, yeast extract 0.5%, MgSO_4_.7H_2_O 0.1%, KH_2_PO_4_ 0.1% in distilled water), static condition, 25 °C, 35 days	*M. tuberculosis* H37Ra	MIC = 25 µg/mL (86.69 µM)	NR	[57]
**71**	palmarumycin P1 [N]	Unidentified marine-derived fungus BCC 250093	Unidentified mangrove wood	Liquid medium (potato dextrose broth), agitated condition at 200 rpm, 25 °C, 20 days	*M. tuberculosis* H37Ra	MIC = 1.56 µg/mL (4.23 µM)	NR	[58]
**72**	palmarumycin P3 [N]					MIC = 12.5 µg/mL (35.28 µM)		
**73**	palmarumycin CP3 [K]					MIC = 1.56 µg/mL (4.64 µM)		
**74**	palmarumycin CR1 [K]					MIC = 3.13 µg/mL (9.36 µM)		
**75**	decaspirone A [K]					MIC = 3.13 µg/mL (9.25 µM)		
**76**	decaspirone C [K]					MIC = 12.5 µg/mL (36.51 µM)		
**77**	oxazinin A [N]	*Xylaria* sp. HDN13-249	Root of mangrove *Sonneratia caseolaris*	Solid medium (soluble starch 4%, yeast extract 0.1%, MgSO_4_ 0.3%, monosodium glutamate 0.2%, sucrose 4%, KH_2_PO_4_ 0.05%, maltose 3%, bean flour 0.05%, peptone 0.2%, agar powder 2.5%, seawater), 28 °C, 30 days	*M. phlei*	MIC = 6.25 µM	NR	[59]
**78**	penixylarin C [N]	*Xylaria* sp. HDN13-249	Root of mangrove *Sonneratia caseolaris*	Solid medium (soluble starch 4%, yeast extract 0.1%, MgSO_4_ 0.3%, monosodium glutamate 0.2%, sucrose 4%, KH_2_PO_4_ 0.05%, maltose 3%, bean flour 0.05%, peptone 0.2%, agar powder 2.5%, seawater), 28 °C, 30 days	*M. phlei*	MIC = 6.25 µM	NR	[60]

* ASW = artificial sea water; ** NA = not active; *** NR = mechanism of action not reported; ^a^ N = novel compound; ^b^ K = known compound.

#### 2.5.2. Peptides and Alkaloids

**Figure 14:**
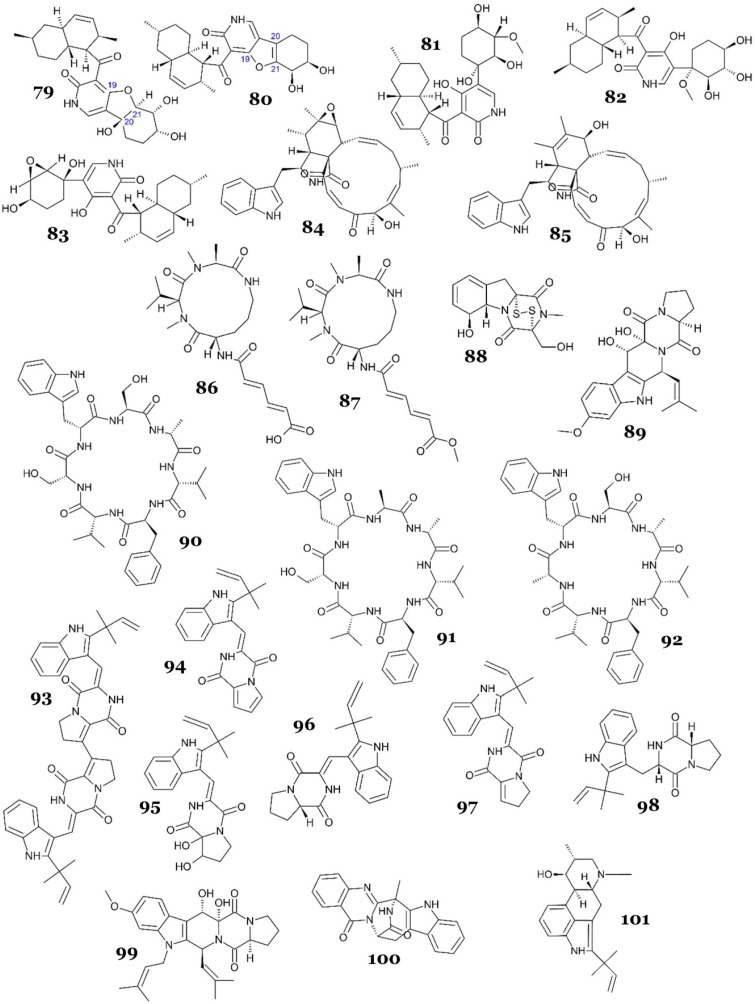
Chemical structures of compounds **79**–**101**.

A collection of novel 4-hydroxy-2-pyridone alkaloids, designated as arthpyrones F-I (**79**–**82**), along with a previously identified compound, apiosporamide (**83**) (Figure 14), were isolated from the deep-sea fungus *Arthrinium* sp. UJNMF008, which was obtained from the South China Sea. Compound **83** strongly inhibited *M. smegmatis*, with an IC_50_ of 2.20 µM, and **79**, **81**, and **82** exhibited moderate to weak antimycobacterial activity, with IC_50_ values in the range of 11.4–35.3 µM, while **80** showed no activity up to 50 µM. Compared with **79**, compound **80** showed a significant loss of activity against *M. smegmatis*, indicating that the hydroxyl at C-20 may be responsible for the activity [61].

Two indole alkaloids, chaetoglobosins A (**84**) and B (**85**) (Figure 14), were re-discovered from the marine alga-derived *Aspergillus fumigatus* AF3-093A collected from the North Atlantic. They displayed antimycobacterial activity against *M. tuberculosis*, with IC_50_ values of 5 and 22 µM, respectively [62]. 

A chemical investigation of the ethyl acetate extract of marine sponge-derived fungus *Aspergillus insulicola* HDN151418 led to the discovery of two new aspochracin-type cyclic tripeptides, sclerotiotides M (**86**) and N (**87**) (Figure 14). Cyclic peptides, crucial metabolites found abundantly in various marine organisms [63,64,65,66,67,68], often feature a distinctive macrocyclic ring and a polyketide side chain in the structure of aspochracin-type cyclic tripeptides. These macrocyclic rings commonly comprise a 12-membered ring (consisting of Ala-Val-Orn) or a 13-membered ring (comprising Ala-Val-Lys). To date, only 15 variants of aspochracin-type cyclic tripeptides have been isolated from their natural origins. A bioactivity assay showed that **86** and **87** exhibited strong to moderate antimycobacterial activity, with MIC values of 3.13 and 12.5 µM, respectively [69]. 

Two diketopiperazine alkaloids with antimycobacterial activity, gliotoxin (**88**) and 12,13-dihydroxy-fumitremorgin C (**89**) (Figure 14), were obtained from the marine-derived *Aspergillus* sp. SCSIO Ind09F01. The fungus was isolated from deep-sea marine sediments (4530 m below sea level) collected from the Indian Ocean. Compounds **88** and **89** demonstrated strong antimycobacterial activity against *M. tuberculosis* H37Ra, with MIC_50_ values of <0.03 and 2.41 µM, respectively. Another compound with a tetracyclic triterpenoid structure, helvolic acid (**116**), also exhibited antimycobacterial properties (this will be described later) [70]. 

Three new cycloheptapeptides, asperversiamides A–C (**90**–**92**) (Figure 14), were successfully discovered from *Aspergillus versicolor* CHNSCLM-0063, isolated from the gorgonian coral *Rumphella aggregata* in South China. Based on the antimycobacterial assay against *M. marinum* and *M. tuberculosis*, all compounds exhibited moderate to weak activity against *M. marinum*, with MICs of 23.4, 81.2, and 87.5 µM, respectively, while only **91** exhibited weak antitubercular activity, with an MIC of 100 µM [71].

Six diketopiperazines were isolated from marine-derived fungus *Aspergillus versicolor* MF030, four of which were new, brevianamides S (**93**), T (**94**), U (**95**), and V (**96**), while the other two were known, brevianamide K (**97**) and deoxybrevianamide E (**98**) (Figure 14). The fungal strain was obtained from marine sediment (60 m below sea level) in the Bohai Sea, northeastern coast of China. All compounds were tested against a panel of microbes, including *M. bovis* BCG. All compounds displayed antimycobacterial activity, with MICs of 6.25, 50, 25, 100, 50, and 100 µg/mL, respectively (9.02, 144.76, 65.54, 286.18, 143.92, and 284.54 µM, respectively). Interestingly, all compounds showed selectivity toward *M. bovis* BCG (no activity against other tested microorganisms up to 100 µg/mL), with compound **93** being the most active against *M. bovis* BCG. This discovery highlights the noteworthy selectivity of dimeric diketopiperazine toward *M. bovis* BCG [72].

*Aspergillus fumigatus* MF071, isolated from marine sediment (60 m below sea level) in the Bohai Sea, China, produced alkaloids fumitremorgin B (**99**), fumiquinazoline J (**100**), and 9-deacetylfumigaclavine C (**101**) (Figure 14), along with terpenoid helvolic acid (**116**), with weak antimycobacterial activity against *M. smegmatis*, with an MIC value of 100 µg/mL (208.52, 280.60, 308.20, and 175.84 µM, respectively) [73]. 

**Figure 15:**
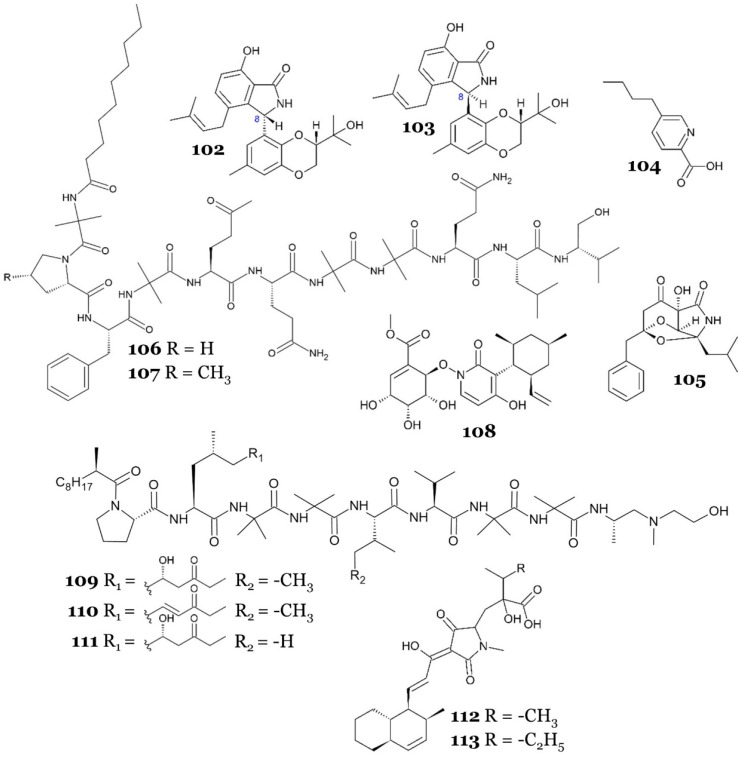
Chemical structures of compounds **102**–**113**.

Two novel isoprenylisoindole alkaloids, diaporisoindoles A (**102**) and B (**103**), were discovered from mangrove-associated fungus *Diaporthe* sp. SYSU-HQ3. Those compounds were the first reported isoprenylisoindole alkaloids with a rare 1,4-benzodioxan moiety. They were tested against MptpB and it was shown that only **102** exhibited potent MptpB inhibitor activity, with an IC_50_ of 4.2 µM, while no inhibitory activity was observed from **103** up to 50 µM. Compounds **102** and **103** differed in the C-8 configuration, 8*S* or 8*R* (Figure 15), which indicated that the 8*S* configuration at C-8 is more beneficial as an MptpB inhibitor. Neither compound showed inhibitory activity against PTP1B up to 200 µM, which showed high selectivity toward MptpB. The kinetic analysis of **102** revealed that it acted as an uncompetitive inhibitor against MptpB. Compound **102** inhibited MptpB with strong activity, whereas its 8 *R* epimer **103** showed only weak activity at 50 µM. Since C-8 defines the relative orientation between the isoindolinone carbonyl, the neighbouring phenolic OH, and the tertiary amine, it is reasonable to propose that the 8 *S* stereochemistry enables these hetero-atoms to face the Lys164/Arg166/Asp165 oxyanion region, whereas the 8 *R* epimer directs them away and may introduce steric interference from the prenyl group. Although this hypothesis fits the observed potency gap, direct structural data are not yet available; hence, further docking or co-crystallography will be required to validate the binding pose. Another terpenoid metabolite, tenellone C (**128**) (which will be described later), also exhibited inhibition against MptpB [74].

A chemical investigation of the ethyl acetate extract of *Fusarium* sp. DZ-27, isolated from *Kandelia cande* (L.) Druce bark, led to the isolation of fusaric acid (**104**). The compound displayed inhibition activity against several mycobacterial strains, including *M. bovis* BCG, *M. tuberculosis* H37Rv, clinical multidrug-resistant *M. tuberculosis* (strain 18019, resistant to SM, INH, RF, and ETH), and clinical multidrug-resistant *M. tuberculosis* (strain 17016, resistant to SM, INH, and ETH), with MIC values of 1.8, 1.8, 30, and 30 µg/mL, respectively (10.04, 10.04, 167.40, and 167.40 µM, respectively) [75]. 

A unique alkaloid, talaramide A (**105**), was isolated from the mangrove endophytic fungus *Talaromyces* sp. HZ-YX1. The fungus strain was obtained from healthy leaves of the mangrove *Kandelia obovata*. The compound displayed antimycobacterial activity by inhibiting the mycobacterial protein kinase G (PknG), a thioredoxin-fold-containing eukaryotic-like serine/threonine protein kinase that acts as a virulence factor of *M. tuberculosis* responsible for the inhibition of phagolysosomal fusion [76], with an IC_50_ value of 55 µM [77]. 

Two novel lipopeptaibols, tolypocaibols A (**106**) and B (**107**), along with the known mixed nonribosomal peptide synthetase (NRPS)–polyketide–shikimate and natural product maximiscin (**108**) (Figure 15), were derived from marine-derived *Tolypocladium* sp. The fungus was isolated from the marine alga *Spongomorpha arcta*, collected from the shores at Green’s Point, L’ Etete, NB, Canada. All compounds were tested against a panel of microorganisms, including *M. smegmatis* and *M. tuberculosis*, and showed antimycobacterial activity, with an MIC of 80 µM against *M. smegmatis* (for compounds **106** and **107**; however, compound **108** was inactive). Antitubercular activity was also observed for all compounds, with MIC values of 20, 40, and 250 µM, respectively [78]. 

Three novel aminolipopeptides, identified as trichoderins A (**109**), A1 (**110**), and B (**111**), were discovered from *Trichoderma* sp., a fungus derived from marine sponge, showcasing antimycobacterial properties effective against both active and dormant bacilli. Compounds **109** and **111** contained the unsual amino acid 2-amino-6-hydroxy-4-methyl-8-oxodecanoic acid (AHMOD) while **110** had 2-amino-4-methy1-8-oxodec-6-enoic acid (AMOD) (Figure 15), which may have affected the antimycobacterial activity of **109**, **110**, and **111**. Those compounds displayed potent antimycobacterial activity against *M. smegmatis*, *M. bovis* BCG, and *M. tuberculosis* in aerobic and hypoxic (dormant state) conditions, with an MIC in the range of 0.02–2 µg/mL. Compounds **109** and **111** exhibited more potent activity (up to 16-fold) against all tested *Mycobacterium* compared with **110**, especially against *M. tuberculosis*. MICs for aerobic and hypoxic conditions for **109** and **111** were 0.12 and 0.13 µg/mL, respectively (103 and 113 nM, respectively), while the MIC for aerobic and hypoxic conditions for **110** was 2.00 µg/mL (1.75 µM) [79]. A further study was conducted to elucidate the possible mechanism of action of **109** by using **109**-resistant clones of *M. smegmatis* mc^2^155. Gene identification on the resistant clones revealed that the target region of **109** contained *atpB*, *atpE*, *atpF*, *atpH*, and parts of *atpA* genes, which are known to express components related to mycobacterial ATP synthesis. This suggested that **109** inhibits mycobacterial cells by inhibiting the mycobacterial ATP synthesis. The prediction was confirmed by using transformants of *M*. *smegmatis* (over-expressed in the regions of *atpB*, *atpE*, *atpF*, and *atpH*), which exhibited resistance in the presence of **109** for up to 0.4 µg/mL, and by measuring the ATP content of *M. bovis* BCG, which showed a reduction of ATP content (80% reduction) in the concentration of 0.1 µg/mL. Trichoderins (**109**–**111**) not only showed activity against dormant mycobacteria but were found to inhibit ATP synthase, hence draining the ATP of non-replicating cells, a mechanism similar to the TB drug bedaquiline [80].

Two novel pyrrolidinone derivatives, zopfiellamides A (**112**) and B (**113**), were discovered from the facultative marine *Zopfiella latipes*, which was obtained from marine sediment in the Indian Ocean. They demonstrated antimycobacterial activity against *M. phlei*, with an MIC in the range of 2–10 µg/mL (4.35–22.44 µM), with **112** being five times more potent than **113** [81].

**Table 2:** Alkaloid and peptide NPs with antimycobacterial activity from MF.

No.	Metabolites [Novelties at the Time of Isolation]	Producing Strains	Marine Sources	Fermentation Media and Method	Tested Against Mycobacterium Strain/Mycobacterial Enzyme	Potency	Mechanism of Action	Ref.
**79**	arthpyrone F [N] ^a^	*Arthrinium* sp. UJNMF008	Marine sediment	Solid medium (80 g commercial rice, 0.4 g yeast extract, 0.4 g glucose, 120 mL water with 3% sea salt), static condition, 28 °C, 30 days	*M. smegmatis*	IC_50_ = 11.4 µM	NR ***	[61]
**80**	arthpyrone G [N]					NA **		
**81**	arthpyrone H [N]					IC_50_ = 19.4 µM		
**82**	arthpyrone I [N]					IC_50_ = 35.3 µM		
**83**	apiosporamide [K] ^b^					IC_50_ = 2.20 µM		
**84**	chaetoglobosin A [N]	*Aspergillus fumigatus* AF3-093A	Marine alga *Fucus vesiculosus*	Liquid medium (malt extract broth 2%), agitated condition at 150 rpm, room temp., 14 days	*M. tuberculosis* H37Ra	MIC = 47 µM	NR	[62]
**85**	chaetoglobosin B [N]					MIC = 95 µM		
**86**	sclerotiotide M [N]	*Aspergillus insulicola* HDN151418	Unidentified marine sponge	Liquid medium (potato dextrose broth), static condition, 28 °C, 30 days	*M. phlei*	MIC = 3.13 µM	NR	[69]
**87**	sclerotiotide N [N]					MIC = 12.5 µM		
**88**	gliotoxin [K]	*Aspergillus* sp. SCSIO Ind09F01	Marine sediment	Liquid medium (mannitol 2%, maltose 2%, glucose 1%, corn steep liquor 0.1%, monosodium glutamate 1%, KH_2_PO_4_ 0.05%, MgSO_4_.7H_2_O 0.03%, yeast extract 0.3%, sea salt 1.5%, pH 7.4), agitated condition at 172 rpm, 27 °C, 15 days	*M. tuberculosis* H37Ra	MIC = <0.03 µM	NR	[70]
**89**	12,13-dihydroxy-fumitremorgin C [K]					MIC = 2.41 µM		
**90**	asperversiamide A [N]	*Aspergillus versicolor CHNSCLM*-0063	Marine gorgonian coral *Rumphella aggregate*	Solid medium (50 g rice, 50 mL sea water), static condition, room temp., 50 days	*M. marinum*	MIC = 23.4 µM	NR	[71]
					*M. tuberculosis*	NA		
**91**	asperversiamide B [N]				*M. marinum*	MIC = 81.2 µM		
					*M. tuberculosis*	MIC = 100 µM		
**92**	asperversiamide C [N]				*M. marinum*	MIC = 87.5 µM		
					*M. tuberculosis*	NA		
**93**	brevianamide S [N]	*Aspergillus versicolor* MF030	Marine sediment	Solid medium (100 g rice, 3.25 g soya bean powder, 30 mL ASW * 3.5%), static condition, 28 °C, 19 days	*M. bovis* BCG	MIC = 6.25 µg/mL (9.02 µM)	NR	[72]
**94**	brevianamide T [N]					MIC = 50 µg/mL (144.76 µM)		
**95**	brevianamide U [N]					MIC = 25 µg/mL (65.54 µM)		
**96**	brevianamide V [N]					MIC = 100 µg/mL (286.18 µM)		
**97**	brevianamide K [K]					MIC = 50 µg/mL (143.92 µM)		
**98**	deoxybrevianamide E [K]					MIC = 100 µg/mL (284.54 µM)		
**99**	fumitremorgin B [K]	*Aspergillus fumigatus* MF071	Marine sediment	Solid medium (160 g rice, 240 mL distilled water), static condition, 28 °C, 30 days	*M. smegmatis*	MIC = 100 µg/mL (208.52 µM)	NR	[73]
**100**	fumiquinazoline J [K]					MIC = 100 µg/mL (280.60µM)		
**101**	9-deacetylfumigaclavine C [K]					MIC = 100 µg/mL (308.20 µM)		
**102**	diaporisoindole A [N]	*Diaporthe* sp. SYSU-HQ3	Branches of mangrove *Excoecaria agallocha*	Solid medium (50 g rice, 50 mL saline water 0.3%), static condition, room temp., 28 days	Mycobacterial Enzyme MptpB	IC_50_ = 4.2 µM	Inhibit virulence factor MptpB	[74]
**103**	diaporisoindole B [N]					NA	-	
**104**	fusaric acid [K]	*Fusarium* sp. DZ-27	Bark of *Kandelia cande* (L.)	Liquid medium (glucose 1%, pepton 0.2 %, yeast extract 0.1%, NaCl 0.3%), static condition, 28 °C, 30 days	*M. bovis* BCG	MIC = 1.8 µg/mL (10.04 µM)	NR	[75]
					*M. tuberculosis* H37Rv	MIC = 1.8 µg/mL (10.04 µM)		
					Clinical multidrug-resistant *M. tuberculosis* (strain 18019, resistant to SM, INH, RF, and ETH)	MIC = 30 µg/mL (167.40 µM)		
					Clinical multidrug-resistant *M. tuberculosis* (strain 17016, resistant to SM, INH, and ETH)	MIC = 30 µg/mL (167.40 µM)		
**105**	talaramide A [N]	*Talaromyces* sp. HZ-YX1	Leaves of mangrove *Kandelia obovata*	Solid medium (50 g rice, 1.5 g artificial sea salts, 50 mL distilled H_2_O), static condition, room temp., 28 days	Mycobacterial Enzyme PknG	IC_50_ = 55 µM	Inhibit virulence factor PknG	[77]
**106**	tolypocaibol A [N]	*Tolypocladium* sp.	Marine alga *Spongomorpha arcta*	Liquid medium (potato dextrose broth 1.2%), agitated condition at 150 rpm, room temp., 14 days	*M. smegmatis* ATCC 70084	MIC = 80 µM	NR	[78]
					*M. tuberculosis* H37Ra	MIC = 20µM		
**107**	tolypocaibol B [N]				*M. smegmatis* ATCC 70084	MIC = 80 µM		
					*M. tuberculosis* H37Ra	MIC = 40 µM		
**108**	maximiscin [K]				*M. smegmatis* ATCC 70084	NA		
					*M. tuberculosis* H37Ra	MIC = 250 µM		
**109**	trichoderin A [N]	*Trichoderma* sp.	Unidentified marine sponge	Solid medium (2.3 kg rice, 4.5 L ASW), static condition, 30 °C, 14 days	*M. smegmatis*	MIC = 0.1 µg/mL (85.95 nM)	Inhibit the mycobacterial F_1_F_0_-ATP–synthase	[79]
					*M. bovis* BCG	MIC = 0.02 µg/mL (17.19 nM)		
					*M. tuberculosis*	MIC = 0.12 µg/mL (103.14 nM)		
**110**	trichoderin A1 [N]				*M. smegmatis*	MIC = 1.56 µg/mL (1.36 µM)		
					*M. bovis* BCG	MIC = 0.16 µg/mL (139.68 nM)		
					*M. tuberculosis*	MIC = 2 µg/mL (1.75 µM)		
**111**	trichoderin B [N]				*M. smegmatis*	MIC = 0.63 µg/mL (548.06 nM)		
					*M. bovis* BCG	MIC = 0.02 µg/mL (17.40 nM)		
					*M. tuberculosis*	MIC = 0.13 µg/mL (113.09 nM)		
**112**	zopfiellamide A [N]	Zopfiella latipes	Marine sediment	Liquid medium (glucose 0.5%, yeast extract 0.1%, peptone from soybean 0.1%, pH 7) in fermentor with aeration rate 3 L/min, 120 rpm, 22 °C, 11 days	*M. phlei*	MIC = 2–10 µg/mL (4.35–22.44 µM)	NR	[81]
**113**	zopfiellamide B [N]					MIC = 2–10 µg/mL (4.35–22.44 µM)		

* ASW = artificial sea water; ** NA = not active; *** NR = mechanism of action not reported; ^a^ N = novel compound; ^b^ K = known compound.

#### 2.5.3. Terpenoids and Steroids

**Figure 16:**
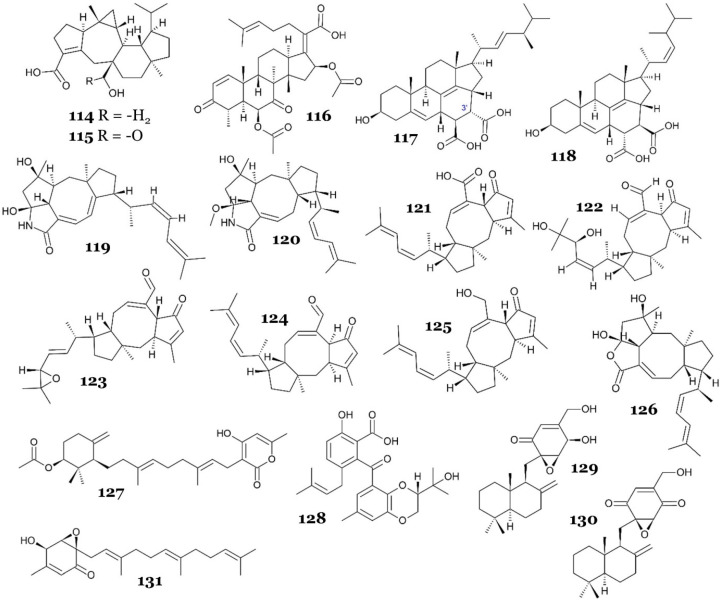
Chemical structures of compounds **114**–**131**.

Asperterpenoid A (**114**), a novel sesterterpenoid with a 5/7/(3)6/5 pentacyclic system (Figure 16), was isolated from the marine-derived fungus *Aspergillus* sp. 16-5c, which was obtained from the mangrove *Sonneratia apetala* collected from the South China Sea. This compound displayed a potent MptpB inhibitor with an IC_50_ of 2.2 µM [82]. In another study, Huang J H and colleagues uncovered a gene cluster comprised of three genes through genome exploration, which was demonstrated to be accountable for the synthesis of asperterpenoid A. The experimental reassembly in *Aspergillus oryzae* NSAR1 unveiled that the terpene synthase AstC functions akin to PvPS, producing preasperterpenoid A. Following this, the P450 AstB-mediated four-step oxidation reactions transformed preasperterpenoid A into the potent MptpB inhibitor **114**, along with a minor byproduct, asperterpenoid B (**115**). The same study also showed that **114** and **115** acted as noncompetitive inhibitors against MptpB, with IC_50_ values of 3.34 and 5.67 µM, respectively, and *K*i values of 2.12 and 2.20 µM, respectively [83]. Asperterpenoids A and B are rigid 5/7/(3)6/5 pentacyclic sesterterpenoids; each bears a carboxylic acid at C-19 and a β-hydroxyl group at C-21, the two polar handles most likely involved in binding to MptpB. Although no co-crystal or docking study is available, the acid/β-OH pair is reminiscent of the multidentate acidic motifs seen in other potent MptpB ligands, suggesting that it may interact with the Lys164/Arg166 oxyanion pocket and/or Asp165, while the bulky terpene cage could fill the hydrophobic groove defined by Phe161 and Tyr125. The modest loss of activity in B may arise from subtle conformational or electronic changes introduced by the epoxide rather than from the addition of a new carbonyl. Further structural data would be required to confirm this binding model [82].

A C-21 steroid, helvolic acid (**116**), was isolated from the marine-derived *Aspergillus* sp. SCSIO Ind09F01, along with **88** and **89**. It exhibited potent antimycobacterial activity against *M. tuberculosis* H37Ra, with an MIC_50_ of 0.894 µM [70].

A chemical investigation of marine-derived *Aspergillus* sp. DM2 ethyl acetate extract led to the discovery of two novel, natural Diels–Alder additive steroids, ergosterdiacids A (**117**) and B (**118**) (Figure 16), with a 6/6/6/6/5 pentacyclic system. An antimycobacterial assay against MptpB showed that **117** and **118** displayed moderate inhibition, with IC_50_ values of 15.1 and 30.1 µM, respectively. According to the docking results, **117** and **118**, alongside oleanolic acid, bound within the active pocket of MptpB, were characterized by amino acid residues of Arg210, Arg63, Arg59, Met206, Phe161, Lle207, Lle203, Phe211, and Glu60. Notably, both compounds displayed interactions between their carboxyl groups and the alkaline amino acids (Arg59, Arg63, Arg210) within the binding pocket, which was reminiscent of (oxalylamino-methylene)–thiophene sulfonamide (OMTS), a potent MptpB inhibitor with an IC_50_ of 440 nM. Specifically, **117** formed four hydrogen bonds in its docking mode: two between the carbonyl carbon of C-3’ and the guanidyl of Arg 210 and Arg63 and two with Arg 63 and Arg59, while **118** exhibited a similar interaction pattern involving the carbonyl carbon of C-3’ and the guanidyl of Arg63 and Arg210. These hydrogen bonds played a pivotal role in stabilizing the protein–ligand complex. Furthermore, the predicted Ki values for **117** and **118** were 267.3 and 34.05 nM, respectively. An in-depth analysis of the structural characteristics of the isolated compounds and OMTS indicated that the carboxyl groups may serve as key functional groups contributing to the inhibitory effects against MptpB [16].

Rai and colleagues discovered 11 new ophiobolin-type sesterterpenoids from mangrove-associated Aspergillus sp. ZJ-68, in which five of them exhibited antimycobacterial properties, asperophiobolins B (**119**), D (**120**), E (**121**), H (**122**), and I (**123**), along with 12 known analogues, three of which, ophiobolin G (**124**), 21-deoxo-21-hydroxy-6-epi-ophiobolin G (**125**), and ophiobolin P (**126**) (Figure 16), also displayed antimycobacterial activity. The assay was conducted against MptpB and revealed that the IC_50_ values of those compounds were in the range of 19–42 µM [84]. Across this ophiobolin/asperophiobolin series, all molecules shared a cavernous 5-8-5 tricyclic cage and a long isoprenyl tail that could fill the hydrophobic Phe161-Leu199 tunnel of MptpB, but provided at most two donors, typically arranged as one dominant lactam/γ-lactone dyad to engage the Lys164/Arg166/Asp165 polar patch, giving uniform, mid-micromolar potencies (IC_50_ = 19–42 µM) [84].

A chemical investigation of an ethyl acetate extract of rice fermentation from the marine-derived *Aspergillus* sp. WHUF03110 led to the isolation of sartopyrone A (**127**). The fungal strain was obtained from the mangrove soil sediment from Yalong Bay, Sanya, Hainan, China. Compound **127** demonstrated moderate activity against *M. smegmatis* ATCC 607, with an MIC of 8 µg/mL (17.52 µM) [85].

A meroterpenoid, tenellone C (**128**), was also isolated from the fungus *Diaporthe* sp. SYSU-HQ3, along with **102** and **103**. Compound **128** was found to be the precursor to **102** and **103**, which could be produced after consecutive reactions of the reduction, nucleophilic addition, and dehydration of **128**. Unlike **102**, compound **128**’s inhibiting property was shown to be a competitive inhibitor against MptpB, with an IC_50_ value of 5.2 µM. However, it had the same selectivity as **102** against MptpB over PtpB [74].

Macrophorin A (**129**), 4′-oxomacrophorin (**130**), and 7-deacetoxyyanuthone A (**131**) were isolated from an ethyl acetate extract of a rice medium of *Gliomastix* sp. obtained from the sponge *Phakellia fusca* Thiele collected in the Yongxing Island of Xisha. Those compounds displayed antimycobacterial activity against *M. tuberculosis* with IC_50_ values of 22.1, 2.44, and 17.5 µM, respectively [86]. 

**Table 3:** Terpenoid and steroid NPs with antimycobacterial activity from MF.

No.	Metabolites [Novelties at the Time of Isolation]	Producing Strains	Marine Sources	Fermentation Media and Method	Tested Against Mycobacterium Strain/Mycobacterial Enzyme	Potency	Mechanism of Action	Ref.
**114**	asperterpenoid A [N] ^a^	*Aspergillus* sp. 16-5c	Mangrove *Sonneratia apetala*	Solid medium (100 g rice, 20 mL 3% sea salt liquid), static condition, 25 °C, 28 days	Mycobacterial Enzyme MptpB	IC_50_ = 3.34 µM *K*i = 2.12 µM	Inhibit virulence factor MptpB	[82,83]
**115**	asperterpenoid B [N]					IC_50_ = 5.67 µM *K*i = 2.20 µM		
**116**	helvolic acid [K] ^b^	*Aspergillus* sp. SCSIO Ind09F01	Marine sediment	Liquid medium (mannitol 2%, maltose 2%, glucose 1%, corn steep liquor 0.1%, monosodium glutamate 1%, KH_2_PO_4_ 0.05%, MgSO_4_.7H_2_O 0.03%, yeast extract 0.3%, sea salt 1.5%, pH 7.4), agitated condition at 172 rpm, 27 °C, 15 days	*M. tuberculosis* H37Ra	MIC_50_ = 0.894 µM	NR *	[70]
**117**	ergosterdiacid A [N]	*Aspergillus* sp. DM2	Mangrove *Aegiceras corniculatum*	Solid medium (50 g corn niblet, 0.86 g yeast extract, 2.37 g ammonium tartrate, 0.17 g MgSO_4_, 0.25 g KH_2_PO_4_, 0.4 g sea salt, 20 mL distilled water), static condition, 28 °C, 20 days	Mycobacterial Enzyme MptpB	IC_50_ = 15.1 µM *K*i = 267.3 nM	Inhibit virulence factor MptpB	[16]
**118**	ergosterdiacid B [N]					IC_50_ = 30.1 µM *K*i = 34.05 nM		
**119**	asperophiobolin B [N]	*Aspergillus* sp. ZJ-68	Leaves of mangrove *Kandelia candel*	Solid medium (50 g rice, 50 mL 0.3% saline water), static condition, 25 °C, 28 days	Mycobacterial Enzyme MptpB	IC_50_ = 39 µM	Inhibit virulence factor MptpB	[84]
**120**	asperophiobolin D [N]					IC_50_ = 42 µM		
**121**	asperophiobolin E [N]					IC_50_ = 28 µM		
**122**	asperophiobolin H [N]					IC_50_ = 19 µM		
**123**	asperophiobolin I [N]					IC_50_ = 35 µM		
**124**	ophiobolin G [K]					IC_50_ = 24 µM		
**125**	21-deoxo-21-hydroxy-6-epi-ophiobolin G [K]					IC_50_ = 37 µM		
**126**	ophiobolin P [K]					IC_50_ = 36 µM		
**127**	sartopyrone A [K]	Aspergillus sp. WHUF03110	Marine sediment	Solid medium (200 g rice, 200 mL distilled water), static condition, 26 °C, 30 days	*M. smegmatis* ATCC 607	MIC = 8 µg/mL (17.52 µM)	NR	[85]
**128**	tenellone C [K]	*Diaporthe* sp. SYSU-HQ3	Branches of mangrove *Excoecaria agallocha*	Solid medium (50 g rice, 50 mL saline water 0.3%), static condition, room temp., 28 days	Mycobacterial Enzyme MptpB	IC_50_ = 5.2 µM	Inhibit virulence factor MptpB	[74]
**129**	macrophorin A [K]	*Gliomastix* sp.	Marine sponge *Phakellia fusca*	Solid medium (200 g rice, 2.5 g sea salt, 200 mL distilled water), static condition, 26 °C, 40 days	*M. tuberculosis*	IC_50_ = 22.1 µM	NR	[86]
**130**	4′-oxomacrophorin [K]					IC_50_ = 2.44 µM		
**131**	7-deacetoxyyanuthone A [K]					IC_50_ = 17.5 µM		

* NR = mechanism of action not reported; ^a^ N = novel compound; ^b^ K = known compound.

#### 2.5.4. Other Compounds

**Figure 17:**
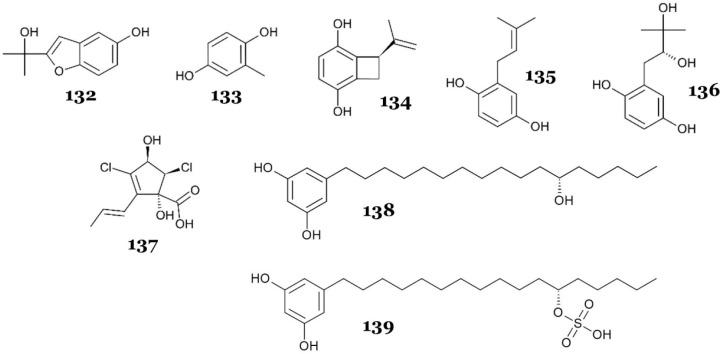
Chemical structures of compounds **132**–**139**.

One novel compound, gliomastin C (**132**), and four known hydroquinone derivatives, methylhydroquinone (**133**), acremonin A (**134**), prenylhydroquinone (**135**), and F-11334A1 (**136**) (Figure 17), were isolated from marine-derived *Gliomastix* sp. The fungus was obtained from the hard coral *Stylophora* sp. (from the Red Sea, Egypt). The compounds displayed antimycobacterial activity against the *M. tuberculosis* strain H37Rv with MICs of 12.5, 12.5, 25, 12.5, and 25 µM, respectively [87].

A novel halogenated metabolite, cryptophomic acid (**137**) (Figure 17), was discovered from marine-derived *Phoma* sp. 135, which was obtained from the sponge *Ectyplasia perox*, collected in Dominica, Lauro Club Reef. It exhibited moderate antimycobacterial activity against *M. phlei*, with an MIC of 16 µM [88].

Two alkyl aromatics, 1,3-dihydroxy-5-(12-hydroxyheptadecyl)benzene (**138**) and 1,3-dihydroxy-5-(12-sulfoxyheptadecyl)benzene (**139**), were isolated from *Xylaria* sp. HDN13-249, along with **77**. The same study also revealed that the yields of those compounds were increased when *Xylaria* sp. HDN13-249 was co-cultivated with the deep-sea fungus *Penicillium crustocum* PRB-2. Compounds **138** and **139** showed antimycobacterial activity against *M. phlei*, with MICs of 25 and 12.5 µM, respectively [60].

**Table 4:** Other NPs with antimycobacterial activity from MF.

No.	Metabolites [Novelties at the Time of Isolation]	Producing Strains	Marine Sources	Fermentation Media and Method	Tested Against Mycobacterium Strain/Mycobacterial Enzyme	Potency	Mechanism of Action	Ref.
**132**	gliomastin C [N] ^a^	*Gliomastix* sp.	Marine coral *Stylophora* sp.	Solid medium (100 g rice, 110 mL water), static condition, 25 °C, 30 days	*M. tuberculosis* H37Rv	MIC = 12.5 µM	NR *	[87]
**133**	methylhydroquinone [K] ^b^					MIC = 12.5 µM		
**134**	acremonin A [K]					MIC = 25 µM		
**135**	prenylhydroquinone [K]					MIC = 12.5 µM		
**136**	F-11334A1 [K]					MIC = 25 µM		
**137**	cryptophomic acid [N]	*Phoma* sp. 135	Marine sponge *Ectyplasia perox*	Solid medium (biomalt agar medium 1.5%), static condition, room temp.	*M. phlei*	MIC = 16 µM	NR	[88]
**138**	1,3-dihydroxy-5-(12-hydroxyheptadecyl)benzene [K]	*Xylaria* sp. HDN13-249	Root of mangrove *Sonneratia caseolaris*	Solid medium (soluble starch 4%, yeast extract 0.1%, MgSO_4_ 0.3%, monosodium glutamate 0.2%, sucrose 4%, KH_2_PO_4_ 0.05%, maltose 3%, bean flour 0.05%, peptone 0.2%, agar powder 2.5%, seawater), 28 °C, 30 days	*M. phlei*	MIC = 25 µM	NR	[60]
**139**	1,3-dihydroxy-5-(12-sulfoxyheptadecyl)benzene [K]					MIC = 12.5 µM		

* NR = mechanism of action not reported; ^a^ N = novel compound; ^b^ K = known compound.

## 3. Perspectives and Outlooks

### 3.1. The Opportunity of Exploring Antimycobacterials from Marine-Derived Fungi

Most of the isolated fungi came from marine sediments, followed by mangrove, marine sponge, and marine algae, with a lesser amount isolated from marine corals, sea fan, ascidian, and marine anemone. This dominance could be attributed to the rich organic matter and unique ecological conditions in sediments, which provide a favorable environment for the growth and metabolic diversification of fungi [89,90]. Mangroves were characterized by their nutrient-rich, brackish waters, and hence can serve as fertile ground for diverse fungal communities [91,92,93]. From this review, it can be seen that marine sponges represent a crucial source of fungi capable of producing bioactive metabolites due to their symbiotic relationships with microorganisms [94]. 

In contrast, marine sources such as algae, corals, sea fans, ascidians, and marine anemones were not well explored in previous studies. This disparity likely reflects a combination of biological and methodological factors, such as their structural and ecological characteristics, or they might have been less extensively sampled in prior studies. While these sources contribute a smaller proportion of fungal isolates, they represent a largely untapped fungal genus that could yield novel compounds, such as compounds **3**, **4**, **35**–**39**, **44**–**45**, **77**, **84**–**85**, **90**–**92**, **132**, **106**, and **107**. This suggests that expanding sampling efforts in corals, sea fans, ascidians, and marine anemones could lead to the discovery of new fungal genera or metabolites.

Our review revealed that *Aspergillus* is the most prevalent species in both marine and terrestrial habitats and produced the most antimycobacterial metabolites, followed by *Penicillium*, making them the most promising sources for the investigation of bioactive metabolites, including potent and novel antimycobacterial agents (compounds **11**, **13**, **55**, **56**, **86**, **114**, and **115**) [16,17,18,19]. However, some less-studied genera, such as *Phoma, Pleosporales, Arthrinium*, *Coniothyrium*, *Talaromyces, Trichoderma*, *Tolycopladium*, and *Zopfiella*, also hold great potential for yielding novel, potent antimycobacterial compounds. For example, *Trichoderma* sp. isolated from unidentified sponge produced novel compounds **109**–**111** that exhibited activity against various *Mycobacterium* strains, not only against the active phenotype but also against the dormant one [79]. *Arthrinium* sp. UJNMF008 produced novel compounds **79**–**82** and known compound **83** with potent activity against *M. smegmatis* [61]. *Zopfiella latipes* from marine sediments produced novel compounds **112**–**113** with potent activity against *M. phlei* [81]. This suggests that exploring antimycobacterials from marine *Aspergillus* and *Penicillium* might be a promising approach due to their abundance in marine environments and ability to produce a variety of active metabolites, but with the possible disadvantage of there being a higher risk of rediscovering known metabolites, or worse, previously reported antimycobacterials. Nonetheless, their lesser discovery was not without reason: their abundance in the environment is, in fact, quite low. Therefore, when attempting to isolate fungi from marine sources, it is recommended to use a larger number of marine samples as one of the strategies to increase the likelihood of isolating fungi from specific genera [19]. 

Finding novel compounds is somewhat crucial in the fight against tuberculosis due to the increased number of drug-resistant strains. Novel compounds may offer alternative mechanisms of action that could potentially overcome existing drug resistance by offering new mechanisms of action. Additionally, novel drugs are hoped to reduce treatment duration and side effects, which could enhance patient compliance and treatment outcomes [95,96]. Since the rediscovery of known compounds remains a significant challenge in the journey of drug discovery from marine resources, integrating dereplication strategies early in the discovery process, such as advanced spectrometric methods, chemoinformatic tools, and genome mining approaches, might help identify previously unknown metabolites [97,98,99]. For example, the use of molecular networking through platforms like GNPS (Global Natural Products Social Molecular Networking) can rapidly dereplicate known compounds, which can save time and resources [100,101]. In addition, combinatorial biosynthesis approaches that combine genetic elements from different fungal species could also diversify the metabolite pool and overcome the limitations of rediscovery [102,103]. Moreover, advances in fermentation optimization and bioprocess engineering, such as the use of co-cultivation strategies, can enhance the metabolic output of marine fungi [104,105].

The research of antituberculosis utilizes not only the *M. tuberculosis* strain but also non-tuberculosis strains such as *M. smegmatis*, *M. bovis* BCG, *M. phlei*, and *M. marinum*. The *M. tuberculosis* strain is a slow-organism and pathogenic organism; hence, research using *M. tuberculosis* must be done in a facility with high safety measures, such as biosafety level 3 (BSL 3), which is not easily available and accessible in most countries [106,107,108]. In addition, research using *M. tuberculosis* requires a long time for practical experiments [106,107,108]. Therefore, most researchers use alternative models with low pathogenicity and rapid growth to investigate antituberculosis activity, but still show comparable susceptibility to *M. tuberculosis*, especially in the screening phase. A study on *M. tuberculosis* genome analysis revealed that about 2800 of 4000 protein-coding genes have comparable equivalents in *M. smegmatis* and *M. bovis* BCG, with >50% similarity in the amino acid composition, suggesting that *M. smegmatis* and *M. bovis* BCG may show comparative susceptibility to *M. tuberculosis* and can be used as a model in the antituberculosis screening process [107]. A successful story of using a non-tuberculosis strain in antituberculosis discovery is the study of bedaquiline, a novel anti-TB drug that acts as a mycobacterial ATP synthase inhibitor that was initially investigated against *M. smegmatis* [109]. Also, the activity spectrum against various *Mycobacterium* strains, such as *M. tuberculosis*, *M. smegmatis*, and *M. bovis* BCG, highlights the utility of surrogate models in early-stage drug discovery. This is particularly relevant for initial screening due to the biosafety and logistical challenges associated with *M. tuberculosis* studies. However, before claiming to be antituberculosis to treat *M. tuberculosis* infection, any potential antimycobacterial hits or leads from screening using *M. smegmatis* or other non-tuberculosis strains must be investigated against *M. tuberculosis* strains eventually. 

As described previously, the need to find sterilizing agents (anti-dormant) that are effective in eradicating tubercles in aerobic and dormant states to shorten the therapy remains critical in antituberculosis discovery [14,36,79,80]. Current TB therapies are time-consuming and ineffective against latent infections, often leading to relapse and contributing to the global disease burden [110,111,112,113]. This review revealed another critical gap in antituberculosis discovery, which is the limited focus on anti-dormancy activity. Dormant *Mycobacterium* populations are key contributors to prolonged tuberculosis therapy, as they exhibit a state of metabolic inactivity that can render them resistant to most conventional antibiotics targeting active bacterial processes [36,79,80]. We found that only 5 out of 131 MF antimycobacterials were tested against non-replicating or dormant phenotypes. This review showed the scarcity of exploring MF NPs and anti-dormancy. To our knowledge, in the last five years, only one study on anti-dormancy from marine-derived fungi has been reported, which is the activity of the ethyl-acetate extract of marine-derived *Aspergillus ostianus* and *Aspergillus flavus* fermentation products against hypoxic-induced dormant *M. smegmatis* [114]. These findings encourage the investigation of MF NPs (previously identified as active against replicating mycobacteria) for their efficacy against dormant *Mycobacterium*. 

### 3.2. Mechanisms of Action of Antimycobacterials

**Figure 18:**
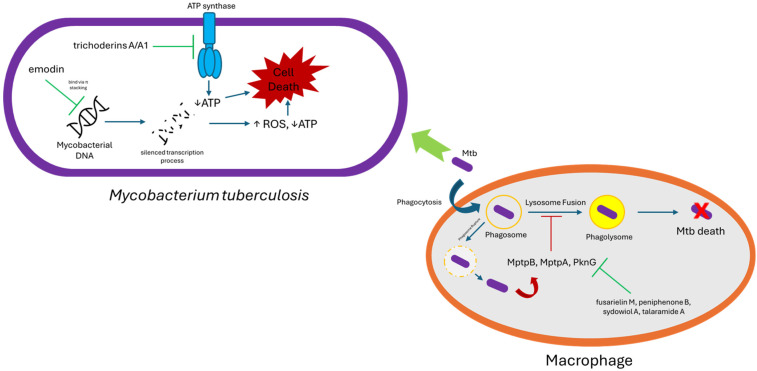
The mechanism of action of several metabolites by inhibiting different targets from *Mycobacterium tuberculosis*.

Marine-derived fungi’s natural products target a remarkably broad spectrum of mycobacterial biochemical pathways, illustrating the power of diverse scaffolds to hit both classical and unconventional targets. A number of isolated metabolites disable mycobacterial virulence factors: for instance, polyketide-derived inhibitors of the secreted protein tyrosine phosphatases MptpB and MptpA can impair the pathogen’s ability to modulate host immune responses. For example, compounds **25**–**27** exemplify this by potently inhibiting MptpB through a distinctive pharmacophore (a rigid 1,4-diketone core flanked by phenolic or β-hydroxy groups) that mimics the phosphate dianion and fits into the enzyme’s wide active site. Other metabolites act on entirely different targets. Compound **105**, a unique alkaloid from *Talaromyces*, inhibits the eukaryotic-like serine/threonine kinase PknG, thereby potentially preventing *M. tuberculosis* from blocking phagosome–lysosome fusion. Likewise, *Trichoderma*-derived aminolipopeptides trichoderins A/A1 (**109**, **110**) and B (**111**) bind the mycobacterial ATP synthase, collapsing ATP generation in non-replicating bacilli in a mechanism analogous to the TB drug bedaquiline. The anthraquinone emodin (**17**) and related planar polyketides offer yet another strategy by intercalating into guanine-rich DNA to stabilize G-quadruplex structures in vital genes (e.g., *mosR* and *ndhA*), thereby suppressing gene expression and slowing growth. This breadth of targets, from central metabolism to DNA topology and virulence signaling, highlights the unique therapeutic promise of marine fungal metabolites in tackling TB from multiple angles. An illustration of the mechanism of action is shown in Figure 18.

### 3.3. Challenges Posed by Mycotoxins

One of the main challenges with drug exploration from fungi, including in marine and terrestrial environments, is their frequent co-classification as mycotoxins or potent cytotoxins. Some of the compounds reported in this article, including those with potent antimycobacterial activity, were reported as mycotoxins and posed significant toxicities to mammals. 

Gliotoxin (**88**) was found to provoke pronounced immunosuppression and lymphocyte apoptosis in mammals, illustrating how potent antimycobacterial efficacy can coexist with unacceptable host toxicity [115]. Secalonic acid D (**34**), while active against *M. tuberculosis*, is simultaneously a teratogenic mycotoxin that induces fetal malformations in rodent models [116]. Helvolic acid (**116**) exerts broad cytotoxic effects on human cancer cell lines and shows limited tolerability in murine in vivo studies [117]. Viomellein (**28**) was reported as a nephrotoxic historically linked to animal mycotoxicoses in contaminated grain, and its structural congener, xanthomegnin (**29**), is both nephrotoxic and mutagenic [118,119]. Tremorgenic indole-diterpenes such as fumitremorgin B (**99**) were found to trigger sustained convulsions, DNA damage in human lymphocytes, and even lethality in mice [120]. Actin-binding cytochalasins including chaetoglobosins A (**84**) and B (**85**) disrupt microfilament polymerization and exhibit potent cytotoxicity toward mammalian cells [121]. Other compounds such as **18**, **104**, **119**, and **124** were found to exhibit cytotoxicity against human cells, neurotoxicity, and immunotoxicity [115,117,122].

Many of the most potent antimycobacterial metabolites isolated from marine fungi carry well-documented liabilities that range from immunosuppression and teratogenicity to nephro- and neurotoxicity in mammalian systems. Their shared propensity for off-target damage suggests a fundamental paradox of fungal natural-product discovery in which structural motifs that confer high antimycobacterial potency often coincide with chemical features that drive host toxicity. Therefore, recognizing these dualities is critical, as it informs the decision of whether a scaffold should be deprioritized, re-engineered, or delivered in a way that limits systemic exposure. 

### 3.4. Anticipating and Mitigating Toxicity in Marine-Derived Fungal Antimycobacterial Discovery

Marine-derived fungi metabolites provide a rich but risk-laden reservoir for antimycobacterial lead discovery. Because many potent hits harbor mycotoxin-associated toxicophores, an effective discovery pipeline must anticipate liabilities as early as dereplication and then deploy a suite of chemical, biosynthetic, pharmacological, and formulation-based tactics to widen the therapeutic window. 

High-resolution LC-MS/MS datasets of an extract can be visualized in GNPS molecular networks or matched in silico to curate the fungal metabolites, hence allowing for the rapid flagging of hazardous chemotypes such as mycotoxins before laborious purification begins [123]. This strategy is also useful as a dereplication tool to avoid re-isolating known fungal metabolites or compounds with previously reported bioactivities [124]. Hence, by overlaying feature-based molecular networking (FBMN) with the public GNPS spectral libraries and in-house mycotoxin databases, investigators can rank network nodes by novelty scores, triage sub-clusters that match hazardous scaffolds, and channel purification resources toward molecular families lacking confident annotations [125]. The FBMN workflow also incorporates quantitative ion-intensity data, enabling the early estimation of compound abundance and signaling when a low-level but novel node may not justify extensive scale-up [125]. Complementary mass-defect filtering and mass-shift searches further sharpen dereplication by excluding spectra that fall within characteristic mass windows of regulated mycotoxins [126,127].

Targeted delivery and formulation-based dose-sparing also offer a complementary route to toxicity mitigation by physically shielding reactive fungal metabolites from off-target tissues and restricting drug exposure to the primary site of infection. Liposomal encapsulation is a clinically validated paradigm that is best exemplified by liposomal amphotericin B, whose reformulation can cut nephro- and infusion-related toxicity relative to the parent macrolide while preserving antifungal potency [128,129]. Similar nanocarrier principles are now being translated to tuberculosis, such as polymeric, lipid, and PLGA nanoparticles that consistently lower the effective doses of rifampicin, isoniazid, and bedaquiline in animal models, thereby reducing systemic adverse effects without compromising bactericidal activity [130,131]. Proof-of-concept for mycotoxins has emerged with magnetic nanoparticle conjugates of gliotoxin, which showed attenuated cytotoxicity in mammalian cells yet retained intracellular antimicrobial activity, demonstrating that nano-shielding can tame even highly reactive species [132,133]. Hence, by applying these orthogonal yet synergistic approaches, researchers can systematically convert hazardous marine-fungal mycotoxins into selective and clinically relevant antitubercular leads.

## 4. Conclusions

Marine-derived fungi (MF) are potential sources of natural products (NPs) with antimycobacterial activity, contributing to a diverse array of metabolites classified into polyketides, alkaloids, peptides, terpenoids, and miscellaneous compounds. Of the 139 identified metabolites, 131 exhibited antimycobacterial activity, with 25 compounds demonstrating strong activity and 50% classified as novel compounds. *Aspergillus* remains the most dominant and productive genus for antimycobacterial compound; however, the utilization of dereplication strategies is necessary to prevent the rediscovery of known antimycobacterials from *Aspergillus*. In addition, focusing on underexplored fungal genera such as *Arthrinium*, *Coniothyrium*, *Zopfiella*, *Trichoderma*, and *Tolypocladium* and targeting marine sources like mangroves, marine sediments, sponges, and algae offer promising avenues for MF antimycobacterial compounds. Nonetheless, the less extensive studies on algae, corals, sea fans, ascidians, and marine anemones suggest that these sources might be a promising source for obtaining novel and unique fungal genera capable of producing bioactive compounds with antimycobacterial activity. Recurrent structural motifs can be discerned, pointing to privileged scaffolds for antimycobacterial activity. For example, several conjugated polyketide frameworks (featuring α,β-unsaturated carbonyls adjacent to hydroxyls) consistently appear in potent isolates, as do planar polyphenolic scaffolds capable of multivalent interactions (e.g., the tri-phenolate arrangement in pyrogallol ethers) and lipophilic cyclic peptides with amphipathic profiles. These pharmacophores underpin interactions with diverse targets, from enzyme active sites to bacterial membranes and DNA, and thus represent valuable starting points for medicinal chemistry optimization. Our SAR analysis across different compound series highlights that even subtle modifications (e.g., the stereochemistry of a double bond or the presence of a specific functional group) can dramatically influence antimycobacterial potency, offering clues for designing analogues with improved efficacy and reduced toxicity. We showed that marine fungal metabolites can inhibit targets and pathways not addressed by current TB drugs, such as virulence factors like MptpB, MptpA, and PknG, and essential bacterial machinery like ATP synthase and DNA topology, thereby opening avenues to overcome existing drug resistance. Compounds acting on such novel targets could retain activity against drug-resistant *M. tuberculosis* strains and even bypass common resistance mechanisms, since their modes of action differ fundamentally from those of first-line agents. Only a small fraction of MF antimycobacterial has been investigated against the dormant phenotype, highlighting a critical gap in current research. These findings underscore the potential of MF as a valuable resource for TB drug discovery, including in addressing drug resistance and dormancy-related challenges.

## References

[1] Barberis I., Bragazzi N.L., Galluzzo L., Martini M. (2017). The History of Tuberculosis: From the First Historical Records to the Isolation of Koch’s Bacillus. J. Prev. Med. Hyg..

[2] World Health Organization (2022). WHO Consolidated Guidelines on Tuberculosis.

[3] Lai P.-C., Low C.-T., Tse W.-S.C., Tsui C.-K., Lee H., Hui P.-K. (2013). Risk of Tuberculosis in High-Rise and High Density Dwellings: An Exploratory Spatial Analysis. Environ. Pollut..

[4] Hunter R.L. (2018). The Pathogenesis of Tuberculosis: The Early Infiltrate of Post-Primary (Adult Pulmonary) Tuberculosis: A Distinct Disease Entity. Front. Immunol..

[5] Rahlwes K.C., Dias B.R.S., Campos P.C., Alvarez-Arguedas S., Shiloh M.U. (2023). Pathogenicity and Virulence of *Mycobacterium tuberculosis*. Virulence.

[6] Smith I. (2003). *Mycobacterium Tuberculosis* Pathogenesis and Molecular Determinants of Virulence. Clin. Microbiol. Rev..

[7] Sigwart J.D., Blasiak R., Jaspars M., Jouffray J.-B., Tasdemir D. (2021). Unlocking the Potential of Marine Biodiscovery. Nat. Prod. Rep..

[8] Carroll A.R., Copp B.R., Davis R.A., Keyzers R.A., Prinsep M.R. (2021). Marine Natural Products. Nat. Prod. Rep..

[9] Cox R.J., Skellam E., Williams K. (2018). Biosynthesis of Fungal Polyketides. Physiology and Genetics.

[10] Harwani D., Barupal S., Begani J., Lakhani J. (2020). Genetic Diversity of Polyketide Synthases and Nonribosomal Peptide Synthetases in Fungi. New and Future Developments in Microbial Biotechnology and Bioengineering.

[11] Rodrigues A.G. (2016). Secondary Metabolism and Antimicrobial Metabolites of Aspergillus. New and Future Developments in Microbial Biotechnology and Bioengineering.

[12] Connolly L.E., Edelstein P.H., Ramakrishnan L. (2007). Why Is Long-Term Therapy Required to Cure Tuberculosis?. PLoS Med..

[13] Sholeye A.R., Williams A.A., Loots D.T., Tutu van Furth A.M., van der Kuip M., Mason S. (2022). Tuberculous Granuloma: Emerging Insights From Proteomics and Metabolomics. Front. Neurol..

[14] Arai M., Kamiya K., Pruksakorn P., Sumii Y., Kotoku N., Joubert J.-P., Moodley P., Han C., Shin D., Kobayashi M. (2015). Anti-Dormant Mycobacterial Activity and Target Analysis of Nybomycin Produced by a Marine-Derived *Streptomyces* Sp. Bioorg. Med. Chem..

[15] Vilchèze C. (2020). Mycobacterial Cell Wall: A Source of Successful Targets for Old and New Drugs. Appl. Sci..

[16] Liu Z., Dong Z., Qiu P., Wang Q., Yan J., Lu Y., Wasu P., Hong K., She Z. (2018). Two New Bioactive Steroids from a Mangrove-Derived Fungus *Aspergillus* Sp. Steroids.

[17] Lee J.W., Lee W., Perera R.H., Lim Y.W. (2023). Long-Term Investigation of Marine-Derived *Aspergillus* Diversity in the Republic of Korea. Mycobiology.

[18] Gonçalves M.F.M., Esteves A.C., Alves A. (2022). Marine Fungi: Opportunities and Challenges. Encyclopedia.

[19] Imhoff J. (2016). Natural Products from Marine Fungi—Still an Underrepresented Resource. Mar. Drugs.

[20] Cos P., Vlietinck A.J., Berghe D.V., Maes L. (2006). Anti-Infective Potential of Natural Products: How to Develop a Stronger In Vitro ‘Proof-of-Concept’. J. Ethnopharmacol..

[21] Xia G., Li J., Li H., Long Y., Lin S., Lu Y., He L., Lin Y., Liu L., She Z. (2014). Alterporriol-Type Dimers from the Mangrove Endophytic Fungus, Alternaria Sp. (SK11), and Their MptpB Inhibitions. Mar. Drugs.

[22] Fan L., Wu X., Jin C., Li F., Xiong S., Dong Y. (2018). MptpB Promotes Mycobacteria Survival by Inhibiting the Expression of Inflammatory Mediators and Cell Apoptosis in Macrophages. Front. Cell Infect. Microbiol..

[23] Vickers C.F., Silva A.P.G., Chakraborty A., Fernandez P., Kurepina N., Saville C., Naranjo Y., Pons M., Schnettger L.S., Gutierrez M.G. (2018). Structure-Based Design of MptpB Inhibitors That Reduce Multidrug-Resistant *Mycobacterium Tuberculosis* Survival and Infection Burden in Vivo. J. Med. Chem..

[24] Seibert S.F., Eguereva E., Krick A., Kehraus S., Voloshina E., Raabe G., Fleischhauer J., Leistner E., Wiese M., Prinz H. (2006). Polyketides from the Marine-Derived Fungus *Ascochyta Salicorniae* and Their Potential to Inhibit Protein Phosphatases. Org. Biomol. Chem..

[25] Zhou B., He Y., Zhang X., Xu J., Luo Y., Wang Y., Franzblau S.G., Yang Z., Chan R.J., Liu Y. (2010). Targeting Mycobacterium Protein Tyrosine Phosphatase B for Antituberculosis Agents. Proc. Natl. Acad. Sci. USA.

[26] Zhang Y., Ling S., Fang Y., Zhu T., Gu Q., Zhu W. (2008). Isolation, Structure Elucidation, and Antimycobacterial Properties of Dimeric Naphtho-*γ*-pyrones from the Marine-Derived Fungus *Aspergillus carbonarius*. Chem. Biodivers..

[27] Liu Z., Wang Q., Li S., Cui H., Sun Z., Chen D., Lu Y., Liu H., Zhang W. (2019). Polypropionate Derivatives with *Mycobacterium Tuberculosis* Protein Tyrosine Phosphatase B Inhibitory Activities from the Deep-Sea-Derived Fungus *Aspergillus Fischeri* FS452. J. Nat. Prod..

[28] Song Z., Liu Y., Gao J., Hu J., He H., Dai S., Wang L., Dai H., Zhang L., Song F. (2021). Antitubercular Metabolites from the Marine-Derived Fungus Strain *Aspergillus Fumigatus* MF029. Nat. Prod. Res..

[29] Dey A., Anand K., Singh A., Prasad R., Barthwal R. (2023). Binding-Induced Thermal Stabilization of MosR and NdhA G-Quadruplex Comprising Genes by Emodin Leads to Downregulation and Growth Inhibition in Mtb: Potential as Anti-Tuberculosis Drug. Results Chem..

[30] Yu G., Wu G., Sun Z., Zhang X., Che Q., Gu Q., Zhu T., Li D., Zhang G. (2018). Cytotoxic Tetrahydroxanthone Dimers from the Mangrove-Associated Fungus Aspergillus Versicolor HDN1009. Mar. Drugs.

[31] Ding L., Ren L., Li S., Song J., Han Z., He S., Xu S. (2019). Production of New Antibacterial 4-Hydroxy-α-Pyrones by a Marine Fungus *Aspergillus Niger.* Cultivated in Solid Medium. Mar. Drugs.

[32] Zhou G., Chen X., Zhang X., Che Q., Zhang G., Zhu T., Gu Q., Li D. (2020). Prenylated *p* -Terphenyls from a Mangrove Endophytic Fungus, *Aspergillus Candidus* LDJ-5. J. Nat. Prod..

[33] Xiao Z., Lin S., Tan C., Lu Y., He L., Huang X., She Z. (2015). Asperlones A and B, Dinaphthalenone Derivatives from a Mangrove Endophytic Fungus Aspergillus Sp. 16-5C. Mar. Drugs.

[34] Beresford N., Patel S., Armstrong J., Szöor B., Fordham-Skelton A.P., Tabernero L. (2007). MptpB, a Virulence Factor from *Mycobacterium Tuberculosis*, Exhibits Triple-Specificity Phosphatase Activity. Biochem. J..

[35] Mascarello A., Mori M., Chiaradia-Delatorre L.D., Menegatti A.C.O., Monache F.D., Ferrari F., Yunes R.A., Nunes R.J., Terenzi H., Botta B. (2013). Discovery of Mycobacterium Tuberculosis Protein Tyrosine Phosphatase B (PtpB) Inhibitors from Natural Products. PLoS ONE.

[36] Kamiya K., Arai M., Setiawan A., Kobayashi M. (2017). Anti-Dormant Mycobacterial Activity of Viomellein and Xanthomegnin, Naphthoquinone Dimers Produced by Marine-Derived *Aspergillus* Sp.. Nat. Prod. Commun..

[37] Gupta V.K., Kumar M.M., Singh D., Bisht D., Sharma S. (2018). Drug Targets in Dormant *Mycobacterium Tuberculosis*: Can the Conquest Against Tuberculosis Become a Reality?. Infect. Dis..

[38] Kovermann M., Stefan A., Palazzetti C., Immler F., Dal Piaz F., Bernardi L., Cimone V., Bellone M.L., Hochkoeppler A. (2023). The *Mycobacterium Tuberculosis* Protein Tyrosine Phosphatase MptpA Features a PH Dependent Activity Overlapping the Bacterium Sensitivity to Acidic Conditions. Biochimie.

[39] Liu X., Song F., Ma L., Chen C., Xiao X., Ren B., Liu X., Dai H., Piggott A.M., Av-Gay Y. (2013). Sydowiols A–C: *Mycobacterium Tuberculosis* Protein Tyrosine Phosphatase Inhibitors from an East China Sea Marine-Derived Fungus, *Aspergillus sydowii*. Tetrahedron Lett..

[40] Stehle T., Sreeramulu S., Löhr F., Richter C., Saxena K., Jonker H.R.A., Schwalbe H. (2012). The Apo-Structure of the Low Molecular Weight Protein-Tyrosine Phosphatase A (MptpA) from Mycobacterium Tuberculosis Allows for Better Target-Specific Drug Development. J. Biol. Chem..

[41] Luo X.-W., Lin Y., Lu Y.-J., Zhou X.-F., Liu Y.-H. (2019). Peptides and Polyketides Isolated from the Marine Sponge-Derived Fungus Aspergillus Terreus SCSIO 41008. Chin. J. Nat. Med..

[42] Wang Y., Lin X.-P., Ju Z.-R., Liao X.-J., Huang X.-J., Zhang C., Zhao B.-X., Xu S.-H. (2017). Aspergchromones A and B, Two New Polyketides from the Marine Sponge-Associated Fungus *Aspergillus* Sp. SCSIO XWS03F03. J. Asian Nat. Prod. Res..

[43] Elsebai M.F., Kehraus S., Lindequist U., Sasse F., Shaaban S., Gütschow M., Josten M., Sahl H.-G., König G.M. (2011). Antimicrobial Phenalenone Derivatives from the Marine-Derived Fungus *Coniothyrium cereale*. Org. Biomol. Chem..

[44] Elsebai M.F., Natesan L., Kehraus S., Mohamed I.E., Schnakenburg G., Sasse F., Shaaban S., Gütschow M., König G.M. (2011). HLE-Inhibitory Alkaloids with a Polyketide Skeleton from the Marine-Derived Fungus *Coniothyrium cereale*. J. Nat. Prod..

[45] Grundner C., Perrin D., Hooft van Huijsduijnen R., Swinnen D., Gonzalez J., Gee C.L., Wells T.N., Alber T. (2007). Structural Basis for Selective Inhibition of *Mycobacterium tuberculosis* Protein Tyrosine Phosphatase PtpB. Structure.

[46] Chen D., Liu L., Lu Y., Chen S. (2021). Identification of Fusarielin M as a Novel Inhibitor of *Mycobacterium Tuberculosis* Protein Tyrosine Phosphatase B (MptpB). Bioorg Chem..

[47] Trisuwan K., Khamthong N., Rukachaisirikul V., Phongpaichit S., Preedanon S., Sakayaroj J. (2010). Anthraquinone, Cyclopentanone, and Naphthoquinone Derivatives from the Sea Fan-Derived Fungi *Fusarium* Spp. PSU-F14 and PSU-F135. J. Nat. Prod..

[48] Kong X., Ma X., Xie Y., Cai S., Zhu T., Gu Q., Li D. (2013). Aromatic Polyketides from a Sponge-Derived Fungus *Metarhizium Anisopliae* Mxh-99 and Their Antitubercular Activities. Arch. Pharm. Res..

[49] Wang C., Wang J., Huang Y., Chen H., Li Y., Zhong L., Chen Y., Chen S., Wang J., Kang J. (2013). Anti-Mycobacterial Activity of Marine Fungus-Derived 4-Deoxybostrycin and Nigrosporin. Molecules.

[50] Sabdaningsih A., Liu Y., Mettal U., Heep J., Riyanti, Wang L., Cristianawati O., Nuryadi H., Triandala Sibero M., Marner M. (2020). A New Citrinin Derivative from the Indonesian Marine Sponge-Associated Fungus Penicillium Citrinum. Mar. Drugs.

[51] Li H., Jiang J., Liu Z., Lin S., Xia G., Xia X., Ding B., He L., Lu Y., She Z. (2014). Peniphenones A–D from the Mangrove Fungus *Penicillium Dipodomyicola* HN4-3A as Inhibitors of *Mycobacterium tuberculosis* Phosphatase MptpB. J. Nat. Prod..

[52] Shah M., Sun C., Sun Z., Zhang G., Che Q., Gu Q., Zhu T., Li D. (2020). Antibacterial Polyketides from Antarctica Sponge-Derived Fungus *Penicillium* Sp. HDN151272. Mar. Drugs.

[53] He F., Li X., Yu J.-H., Zhang X., Nong X., Chen G., Zhu K., Wang Y.-Y., Bao J., Zhang H. (2019). Secondary Metabolites from the Mangrove Sediment-Derived Fungus *Penicillium Pinophilum* SCAU037. Fitoterapia.

[54] Morehouse N.J., Flewelling A.J., Johnson J.A., Gray C.A. (2020). Halogenated Bianthrones from *Penicillium Roseopurpureum*: A Fungal Endophyte of the Marine Alga *Petalonia fascia*. Nat. Prod. Commun..

[55] Han Y., Sun C., Li C., Zhang G., Zhu T., Li D., Che Q. (2021). Antibacterial Phenalenone Derivatives from Marine-Derived Fungus *Pleosporales* Sp. HDN1811400. Tetrahedron Lett..

[56] Ge X., Sun C., Feng Y., Wang L., Peng J., Che Q., Gu Q., Zhu T., Li D., Zhang G. (2019). Anthraquinone Derivatives from a Marine-Derived Fungus *Sporendonema Casei* HDN16-802. Mar. Drugs.

[57] Chokpaiboon S., Unagul P., Nithithanasilp S., Komwijit S., Somyong W., Ratiarpakul T., Isaka M., Bunyapaiboonsri T. (2018). Salicylaldehyde and Dihydroisobenzofuran Derivatives from the Marine Fungus *Zopfiella marina*. Nat. Prod. Res..

[58] Bunyapaiboonsri T., Yoiprommarat S., Nopgason R., Intereya K., Suvannakad R., Sakayaroj J. (2015). Palmarumycins from the Mangrove Fungus BCC 25093. Tetrahedron.

[59] Lin Z., Koch M., Abdel Aziz M.H., Galindo-Murillo R., Tianero M.D., Cheatham T.E., Barrows L.R., Reilly C.A., Schmidt E.W. (2014). Oxazinin A, a Pseudodimeric Natural Product of Mixed Biosynthetic Origin from a Filamentous Fungus. Org. Lett..

[60] Yu G., Sun Z., Peng J., Zhu M., Che Q., Zhang G., Zhu T., Gu Q., Li D. (2019). Secondary Metabolites Produced by Combined Culture of *Penicillium crustosum* and a *Xylaria* Sp. J. Nat. Prod..

[61] Bao J., Zhai H., Zhu K., Yu J.-H., Zhang Y., Wang Y., Jiang C.-S., Zhang X., Zhang Y., Zhang H. (2018). Bioactive Pyridone Alkaloids from a Deep-Sea-Derived Fungus *Arthrinium* Sp. UJNMF0008. Mar. Drugs.

[62] Flewelling A.J., Bishop A.L., Johnson J.A., Gray C.A. (2015). Polyketides from an Endophytic *Aspergillus Fumigatus* Isolate Inhibit the Growth of *Mycobacterium Tuberculosis* and MRSA. Nat. Prod. Commun..

[63] Zheng J., Xu Z., Wang Y., Hong K., Liu P., Zhu W. (2010). Cyclic Tripeptides from the Halotolerant Fungus *Aspergillus Sclerotiorum* PT06-1. J. Nat. Prod..

[64] Vellé A., Cebollada A., Macías R., Iglesias M., Gil-Moles M., Sanz Miguel P.J. (2017). From Imidazole toward Imidazolium Salts and N-Heterocyclic Carbene Ligands: Electronic and Geometrical Redistribution. ACS Omega.

[65] Wu Y., Liao H., Liu L.-Y., Sun F., Chen H.-F., Jiao W.-H., Zhu H.-R., Yang F., Huang G., Zeng D.-Q. (2020). Phakefustatins A–C: Kynurenine-Bearing Cycloheptapeptides as RXRα Modulators from the Marine Sponge *Phakellia fusca*. Org. Lett..

[66] Xu W.-J., Liao X.-J., Xu S.-H., Diao J.-Z., Du B., Zhou X.-L., Pan S.-S. (2008). Isolation, Structure Determination, and Synthesis of Galaxamide, A Rare Cytotoxic Cyclic Pentapeptide from a Marine Algae *Galaxaura filamentosa*. Org. Lett..

[67] Teta R., Marteinsson V.T., Longeon A., Klonowski A.M., Groben R., Bourguet-Kondracki M.-L., Costantino V., Mangoni A. (2017). Thermoactinoamide A, an Antibiotic Lipophilic Cyclopeptide from the Icelandic Thermophilic Bacterium *Thermoactinomyces vulgaris*. J. Nat. Prod..

[68] Skehan P., Storeng R., Scudiero D., Monks A., McMahon J., Vistica D., Warren J.T., Bokesch H., Kenney S., Boyd M.R. (1990). New Colorimetric Cytotoxicity Assay for Anticancer-Drug Screening. JNCI J. Natl. Cancer Inst..

[69] Sun C., Zhang Z., Ren Z., Yu L., Zhou H., Han Y., Shah M., Che Q., Zhang G., Li D. (2020). Antibacterial Cyclic Tripeptides from Antarctica-Sponge-Derived Fungus *Aspergillus Insulicola* HDN151418. Mar. Drugs.

[70] Luo X., Zhou X., Lin X., Qin X., Zhang T., Wang J., Tu Z., Yang B., Liao S., Tian Y. (2017). Antituberculosis Compounds from a Deep-Sea-Derived Fungus *Aspergillus* Sp. SCSIO Ind09F01. Nat. Prod. Res..

[71] Hou X.-M., Liang T.-M., Guo Z.-Y., Wang C.-Y., Shao C.-L. (2019). Discovery, Absolute Assignments, and Total Synthesis of Asperversiamides A–C and Their Potent Activity Against *Mycobacterium marinum*. Chem. Commun..

[72] Song F., Liu X., Guo H., Ren B., Chen C., Piggott A.M., Yu K., Gao H., Wang Q., Liu M. (2012). Brevianamides with Antitubercular Potential from a Marine-Derived Isolate of *Aspergillus versicolor*. Org. Lett..

[73] Han J., Liu M., Jenkins I.D., Liu X., Zhang L., Quinn R.J., Feng Y. (2020). Genome-Inspired Chemical Exploration of Marine Fungus *Aspergillus Fumigatus* MF071. Mar. Drugs.

[74] Cui H., Lin Y., Luo M., Lu Y., Huang X., She Z. (2017). Diaporisoindoles A–C: Three Isoprenylisoindole Alkaloid Derivatives from the Mangrove Endophytic Fungus *Diaporthe* Sp. SYSU-HQ3. Org. Lett..

[75] Pan J.-H., Chen Y., Huang Y.-H., Tao Y.-W., Wang J., Li Y., Peng Y., Dong T., Lai X.-M., Lin Y.-C. (2011). Antimycobacterial Activity of Fusaric Acid From a Mangrove Endophyte and Its Metal Complexes. Arch. Pharm. Res..

[76] Khan M.Z., Bhaskar A., Upadhyay S., Kumari P., Rajmani R.S., Jain P., Singh A., Kumar D., Bhavesh N.S., Nandicoori V.K. (2017). Protein Kinase G Confers Survival Advantage to *Mycobacterium Tuberculosis* during Latency-Like Conditions. J. Biol. Chem..

[77] Chen S., He L., Chen D., Cai R., Long Y., Lu Y., She Z. (2017). Talaramide A, an Unusual Alkaloid From The Mangrove Endophytic Fungus *Talaromyces* Sp. (HZ-YX1) as an Inhibitor of Mycobacterial PknG. New J. Chem..

[78] Morehouse N.J., Flewelling A.J., Liu D.Y., Cavanagh H., Linington R.G., Johnson J.A., Gray C.A. (2023). Tolypocaibols: Antibacterial Lipopeptaibols from a *Tolypocladium* Sp. Endophyte of the Marine Macroalga *Spongomorpha arcta*. J. Nat. Prod..

[79] Pruksakorn P., Arai M., Kotoku N., Vilchèze C., Baughn A.D., Moodley P., Jacobs W.R., Kobayashi M. (2010). Trichoderins, Novel Aminolipopeptides from a Marine Sponge-Derived Trichoderma Sp., Are Active Against Dormant Mycobacteria. Bioorg. Med. Chem. Lett..

[80] Pruksakorn P., Arai M., Liu L., Moodley P., Jacobs W.R., Kobayashi M. (2011). Action-Mechanism of Trichoderin A, an Anti-Dormant Mycobacterial Aminolipopeptide from Marine Sponge-Derived *Trichoderma* Sp. Biol. Pharm. Bull..

[81] Daferner M., Anke T., Sterner O. (2002). Zopfiellamides A and B, Antimicrobial Pyrrolidinone Derivatives from the Marine Fungus *Zopfiella latipes*. Tetrahedron.

[82] Huang X., Huang H., Li H., Sun X., Huang H., Lu Y., Lin Y., Long Y., She Z. (2013). Asperterpenoid A, a New Sesterterpenoid as an Inhibitor of *Mycobacterium Tuberculosi* s Protein Tyrosine Phosphatase B from the Culture of *Aspergillus* Sp. 16-5c. Org. Lett..

[83] Huang J.-H., Lv J.-M., Wang Q.-Z., Zou J., Lu Y.-J., Wang Q.-L., Chen D.-N., Yao X.-S., Gao H., Hu D. (2019). Biosynthesis of an Anti-Tuberculosis Sesterterpenoid Asperterpenoid A. Org. Biomol. Chem..

[84] Cai R., Jiang H., Mo Y., Guo H., Li C., Long Y., Zang Z., She Z. (2019). Ophiobolin-Type Sesterterpenoids from the Mangrove Endophytic Fungus *Aspergillus* Sp. ZJ-68. J. Nat. Prod..

[85] Lv H., Wang K., Xue Y., Chen J., Su H., Zhang J., Wu Y., Jia J., Bi H., Wang H. (2021). Three New Metabolites From the Marine-Derived Fungus *Aspergillus* Sp. WHUF03110. Nat. Prod. Commun..

[86] He W.-J., Zhou X.-J., Qin X.-C., Mai Y.-X., Lin X.-P., Liao S.-R., Yang B., Zhang T., Tu Z.-C., Wang J.-F. (2017). Quinone/Hydroquinone Meroterpenoids with Antitubercular and Cytotoxic Activities Produced by the Sponge-Derived Fungus *Gliomastix* Sp. ZSDS1-F7. Nat. Prod. Res..

[87] Elnaggar M.S., Ebrahim W., Mándi A., Kurtán T., Müller W.E.G., Kalscheuer R., Singab A., Lin W., Liu Z., Proksch P. (2017). Hydroquinone Derivatives from the Marine-Derived Fungus *Gliomastix* Sp. RSC Adv..

[88] Elsebai M.F., Ghabbour H.A., Legrave N., Fontaine-Vive F., Mehiri M. (2018). New Bioactive Chlorinated Cyclopentene Derivatives from the Marine-Derived Fungus *Phoma* Sp. Med. Chem. Res..

[89] Amend A., Burgaud G., Cunliffe M., Edgcomb V.P., Ettinger C.L., Gutiérrez M.H., Heitman J., Hom E.F.Y., Ianiri G., Jones A.C. (2019). Fungi in the Marine Environment: Open Questions and Unsolved Problems. mBio.

[90] Sen K., Sen B., Wang G. (2022). Diversity, Abundance, and Ecological Roles of Planktonic Fungi in Marine Environments. J. Fungi.

[91] Akram H., Hussain S., Mazumdar P., Chua K.O., Butt T.E., Harikrishna J.A. (2023). Mangrove Health: A Review of Functions, Threats, and Challenges Associated with Mangrove Management Practices. Forests.

[92] Augusthy S., Nizam A., Kumar A. (2024). The Diversity, Drivers, Consequences and Management of Plant Invasions in the Mangrove Ecosystems. Sci. Total Environ..

[93] Thatoi H., Behera B.C., Mishra R.R. (2013). Ecological Role and Biotechnological Potential of Mangrove Fungi: A Review. Mycology.

[94] Kushveer J.S., Rashmi M., Sarma V.V. (2021). Bioactive Compounds from Marine-Derived Fungi and Their Potential Applications. Fungi Bio-Prospects in Sustainable Agriculture, Environment and Nano-Technology.

[95] Lienhardt C., Raviglione M., Spigelman M., Hafner R., Jaramillo E., Hoelscher M., Zumla A., Gheuens J. (2012). New Drugs for the Treatment of Tuberculosis: Needs, Challenges, Promise, and Prospects for the Future. J. Infect. Dis..

[96] Motta I., Boeree M., Chesov D., Dheda K., Günther G., Horsburgh C.R., Kherabi Y., Lange C., Lienhardt C., McIlleron H.M. (2024). Recent Advances in the Treatment of Tuberculosis. Clin. Microbiol. Infect..

[97] Albarano L., Esposito R., Ruocco N., Costantini M. (2020). Genome Mining as New Challenge in Natural Products Discovery. Mar. Drugs.

[98] Gaudêncio S.P., Pereira F. (2015). Dereplication: Racing to Speed up the Natural Products Discovery Process. Nat. Prod. Rep..

[99] Tawfike A.F., Viegelmann C., Edrada-Ebel R. (2013). Metabolomics and Dereplication Strategies in Natural Products. Metabolomics Tools for Natural Product Discovery: Methods and Protocols.

[100] Qin G.-F., Zhang X., Zhu F., Huo Z.-Q., Yao Q.-Q., Feng Q., Liu Z., Zhang G.-M., Yao J.-C., Liang H.-B. (2022). MS/MS-Based Molecular Networking: An Efficient Approach for Natural Products Dereplication. Molecules.

[101] Wang M., Carver J.J., Phelan V.V., Sanchez L.M., Garg N., Peng Y., Nguyen D.D., Watrous J., Kapono C.A., Luzzatto-Knaan T. (2016). Sharing and Community Curation of Mass Spectrometry Data with Global Natural Products Social Molecular Networking. Nat. Biotechnol..

[102] Jiang C., Lv G., Tu Y., Cheng X., Duan Y., Zeng B., He B. (2021). Applications of CRISPR/Cas9 in the Synthesis of Secondary Metabolites in Filamentous Fungi. Front. Microbiol..

[103] Skellam E., Rajendran S., Li L. (2024). Combinatorial Biosynthesis for the Engineering of Novel Fungal Natural Products. Commun. Chem..

[104] Naseema Rasheed R., Pourbakhtiar A., Mehdizadeh Allaf M., Baharlooeian M., Rafiei N., Alishah Aratboni H., Morones-Ramirez J.R., Winck F.V. (2023). Microalgal Co-Cultivation-Recent Methods, Trends in Omic-Studies, Applications, and Future Challenges. Front. Bioeng. Biotechnol..

[105] Oppong-Danquah E., Blümel M., Scarpato S., Mangoni A., Tasdemir D. (2022). Induction of Isochromanones by Co-Cultivation of the Marine Fungus Cosmospora Sp. and the Phytopathogen Magnaporthe Oryzae. Int. J. Mol. Sci..

[106] Mtafya B., Musisi E., Qwaray P., Sichone E., Walbaum N., Ntinginya N.E., Gillespie S.H., Sabiiti W. (2024). Quantifying Viable M. Tuberculosis Safely Obviating the Need for High Containment Facilities. Methods Mol. Biol..

[107] Gordon S.V., Parish T. (2018). Microbe Profile: Mycobacterium Tuberculosis: Humanity’s Deadly Microbial Foe. Microbiology.

[108] Baker J.J., Johnson B.K., Abramovitch R.B. (2014). Slow Growth of *Mycobacterium tuberculosis* at Acidic pH Is Regulated by *PhoPR* and Host-associated Carbon Sources. Mol. Microbiol..

[109] Andries K., Verhasselt P., Guillemont J., Göhlmann H.W.H., Neefs J.-M., Winkler H., Van Gestel J., Timmerman P., Zhu M., Lee E. (2005). A Diarylquinoline Drug Active on the ATP Synthase of *Mycobacterium tuberculosis*. Science.

[110] Dutta N.K., Karakousis P.C. (2014). Latent Tuberculosis Infection: Myths, Models, and Molecular Mechanisms. Microbiol. Mol. Biol. Rev..

[111] Magombedze G., Dowdy D., Mulder N. (2013). Latent Tuberculosis: Models, Computational Efforts and the Pathogen’s Regulatory Mechanisms during Dormancy. Front. Bioeng. Biotechnol..

[112] Ahmad S. (2011). Pathogenesis, Immunology, and Diagnosis of Latent *Mycobacterium Tuberculosis* Infection. Clin. Dev. Immunol..

[113] Lin P.L., Flynn J.L. (2010). Understanding Latent Tuberculosis: A Moving Target. J. Immunol..

[114] Azhari M., Litanjuasari A.P., Singgih M., Arai M., Handayani D., Artasasta M.A., Julianti E. (2025). Activity of Ethyl Acetate Extracts of Marine-Derived Fungi against Active and Hypoxia-Induced Dormant Mycobacterium. J. Pharm. Pharmacogn. Res..

[115] Pahl H.L., Krauss B., Schulze-Osthoff K., Decker T., Traenckner E.B., Vogt M., Myers C., Parks T., Warring P., Mühlbacher A. (1996). The Immunosuppressive Fungal Metabolite Gliotoxin Specifically Inhibits Transcription Factor NF-KappaB. J. Exp. Med..

[116] Mayura K., Wallace Hayes A., Berndt W.O. (1982). Teratogenicity of Secalonic Acid d in Rats. Toxicology.

[117] Xiao J.-H., Zhang Y., Liang G.-Y., Liu R.-M., Li X.-G., Zhang L.-T., Chen D.-X., Zhong J.-J. (2017). Synergistic Antitumor Efficacy of Antibacterial Helvolic Acid from *Cordyceps Taii* and Cyclophosphamide in a Tumor Mouse Model. Exp. Biol. Med..

[118] Hald B., Christensen D.H., Krogh P. (1983). Natural Occurrence of the Mycotoxin Viomellein in Barley and the Associated Quinone-Producing Penicillia. Appl. Env. Microbiol..

[119] Gupta A.K., Ahmad I., Borst I., Summerbell R.C. (2000). Detection of Xanthomegnin in Epidermal Materials Infected with Trichophyton Rubrum. J. Investig. Dermatol..

[120] Sabater-Vilar M., Nijmeijer S., Fink-Gremmels J. (2003). Genotoxicity Assessment of Five Tremorgenic Mycotoxins (Fumitremorgen B, Paxilline, Penitrem A, Verruculogen, and Verrucosidin) Produced by Molds Isolated from Fermented Meats. J. Food Prot..

[121] Lambert C., Schmidt K., Karger M., Stadler M., Stradal T.E.B., Rottner K. (2023). Cytochalasans and Their Impact on Actin Filament Remodeling. Biomolecules.

[122] Gauthier T., Wang X., Sifuentes Dos Santos J., Fysikopoulos A., Tadrist S., Canlet C., Artigot M.P., Loiseau N., Oswald I.P., Puel O. (2012). Trypacidin, a Spore-Borne Toxin from Aspergillus Fumigatus, Is Cytotoxic to Lung Cells. PLoS ONE.

[123] Villanueva-Silva R., Velez P., Riquelme M., Fajardo-Hernández C.A., Martínez-Cárdenas A., Arista-Romero A., Wan B., Ma R., Qader M., Franzblau S.G. (2021). Chemical Diversity and Antimicrobial Potential of Cultivable Fungi from Deep-Sea Sediments of the Gulf of Mexico. Molecules.

[124] Chen M., Hao B.-C., Zhu X.-H., Zhang L.-K., Zheng Y.-Y., Zhou X.-J., Schäberle T.F., Shen L., Wang C.-Y., Liu Y. (2025). Molecular Networking Reveals Indole Diterpenoids from the Marine-Derived Fungus Penicillium Sp. N4-3. Mar. Life Sci. Technol..

[125] Nothias L.-F., Petras D., Schmid R., Dührkop K., Rainer J., Sarvepalli A., Protsyuk I., Ernst M., Tsugawa H., Fleischauer M. (2020). Feature-Based Molecular Networking in the GNPS Analysis Environment. Nat. Methods.

[126] Paguigan N.D., El-Elimat T., Kao D., Raja H.A., Pearce C.J., Oberlies N.H. (2017). Enhanced Dereplication of Fungal Cultures via Use of Mass Defect Filtering. J. Antibiot..

[127] Gao Y., Wang Y., Chung H., Chen K., Shen T., Hsu C. (2020). Molecular Networking as a Dereplication Strategy for Monitoring Metabolites of Natural Product Treated Cancer Cells. Rapid Commun. Mass Spectrom..

[128] Roberts J., Bingham J., McLaren A.C., McLemore R. (2015). Liposomal Formulation Decreases Toxicity of Amphotericin B In Vitro and In Vivo. Clin. Orthop. Relat. Res..

[129] Faustino C., Pinheiro L. (2020). Lipid Systems for the Delivery of Amphotericin B in Antifungal Therapy. Pharmaceutics.

[130] Kumar M., Virmani T., Kumar G., Deshmukh R., Sharma A., Duarte S., Brandão P., Fonte P. (2023). Nanocarriers in Tuberculosis Treatment: Challenges and Delivery Strategies. Pharmaceuticals.

[131] Shao L., Shen S., Liu H. (2022). Recent Advances in PLGA Micro/Nanoparticle Delivery Systems as Novel Therapeutic Approach for Drug-Resistant Tuberculosis. Front. Bioeng. Biotechnol..

[132] Comas L., Polo E., Domingo M., Hernández Y., Arias M., Esteban P., Martínez-Lostao L., Pardo J., Martínez de la Fuente J., Gálvez E. (2019). Intracellular Delivery of Biologically-Active Fungal Metabolite Gliotoxin Using Magnetic Nanoparticles. Materials.

[133] Ye W., Liu T., Zhang W., Zhang W. (2021). The Toxic Mechanism of Gliotoxins and Biosynthetic Strategies for Toxicity Prevention. Int. J. Mol. Sci..

